# Supplementary material for: Wastewater surveillance reveals patterns of antibiotic resistance across the United States
Source: Nat Commun. 2026 Apr 1;17:4680. doi: 10.1038/s41467-026-71195-4 (PMC13201604; doi:10.1038/s41467-026-71195-4)
Supplement: Supplementary file 1 — Supplementary Information [file 41467_2026_71195_MOESM1_ESM.pdf]

Supplementary Information

Wastewater surveillance reveals patterns of antibiotic resistance across the United States

Authors: Sooyeol Kim[1], Alessandro Zulli[2], Elana M. G. Chan[2], Dorothea Duong[3], Rebecca Y. Linfield[4], Caroline E. M. McCormack[1], Bradley J. White[3,5], Marlene K. Wolfe[6], Alexandria B. Boehm[2], Amy J. Pickering*[1,7,8]

*Corresponding author: pickering@berkeley.edu

Affiliation
[1]Department of Civil and Environmental Engineering, University of California, Berkeley, Berkeley, CA, 94720, USA
[2]Department of Civil and Environmental Engineering, Stanford University, Stanford, CA, 94305, USA
[3]Verily Life Sciences, South San Francisco, CA, 94080, USA
[4]Division of Infectious Diseases and Geographic Medicine, Department of Medicine, Stanford University, Stanford, CA, 94305, USA
[5]Google LLC, Mountain View, CA, 94043, USA
[6]Gangarosa Department of Environmental Health, Rollins School of Public Health, Emory University, Atlanta, GA, 30329, USA
[7]Chan Zuckerberg Biohub San Francisco, San Francisco, CA, 94158, USA
[8]Blum Center for Developing Economies, University of California, Berkeley, Berkeley, CA, 94720, USA

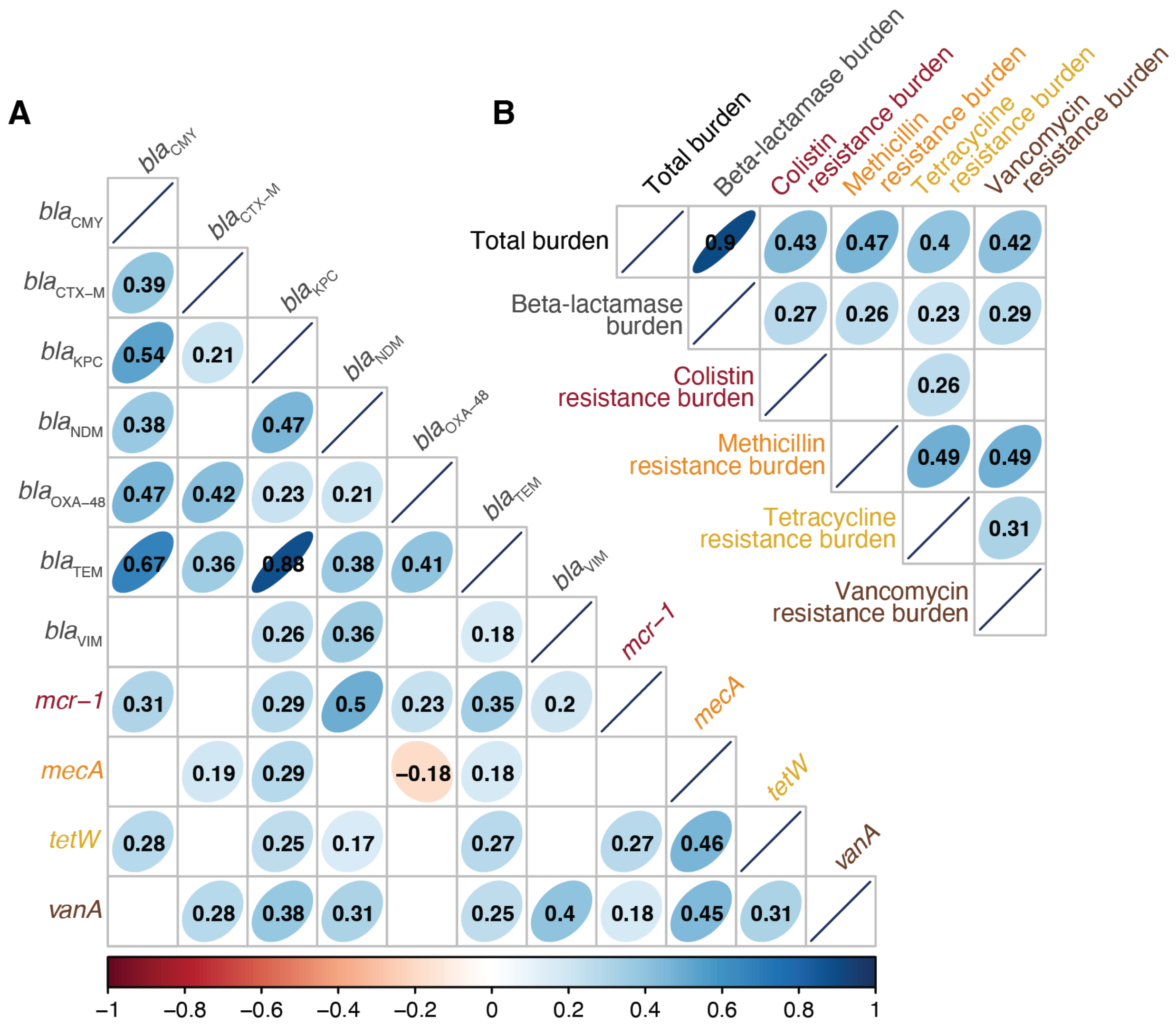

Figure S1. **Correlation among ARGs measured in wastewater. A)** One-sided Spearman's correlation coefficient among ARG concentrations normalized by 16S rRNA gene ($p < 0.05$). **B)** One-sided Spearman's correlation coefficient among antibiotic resistance burden calculated as z-scores based on distribution of each gene ($p < 0.05$).

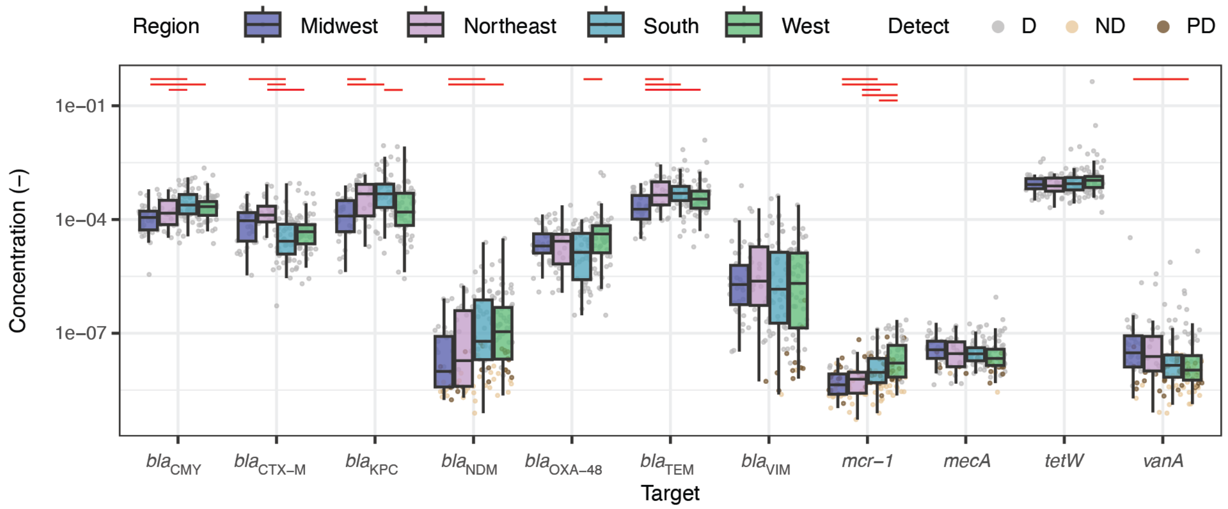

Figure S2. Regional distribution of all ARGs. Each data point represents a wastewater treatment plant (n = 32 for Midwest, n = 24 for Northeast, n = 48 for South, and n = 59 for West). Samples were categorized as non-detect (ND) if none of the replicates detected the target, detect (D) if all replicates detected it, and partial detect (PD) if at least one of the replicates did not detect the target. If the gene was undetected, half of the theoretical measurement limit was substituted as the measured value. The median is shown by the line inside the box with the 25th and 75th percentile represented by the lower and upper boundary of the box. Bottom and top whiskers show 1.5 x interquartile range. Red lines indicate a significant pairwise difference between regions measured by the two-tail Conover-Iman post-hoc test with Benjamini-Hochberg correction applied ($p < 0.025$).

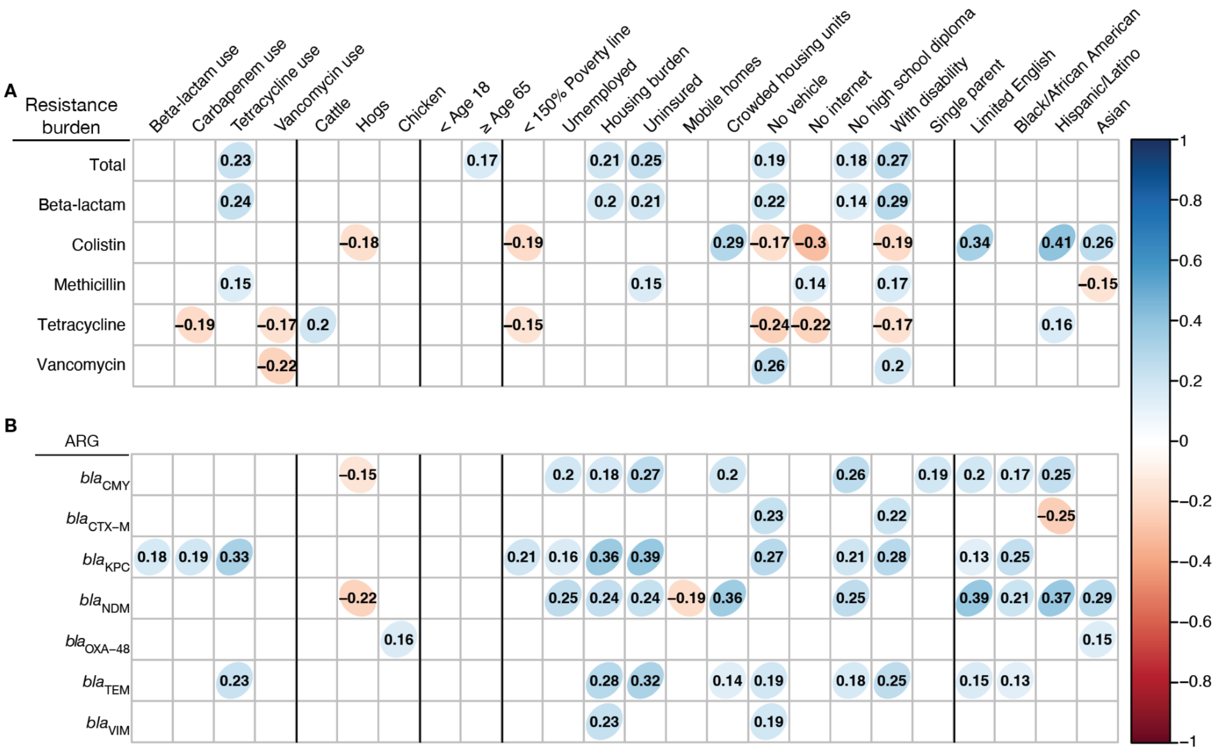

Figure S3. Unadjusted correlation resistance measured in wastewater and potential determinants. One-sided Spearman correlation coefficient among potential determinants of resistance burden and **A)** antibiotic resistance burden score and **B)** beta-lactamase gene concentrations measured in wastewater and normalized by 16S rRNA gene ($p < 0.05$).

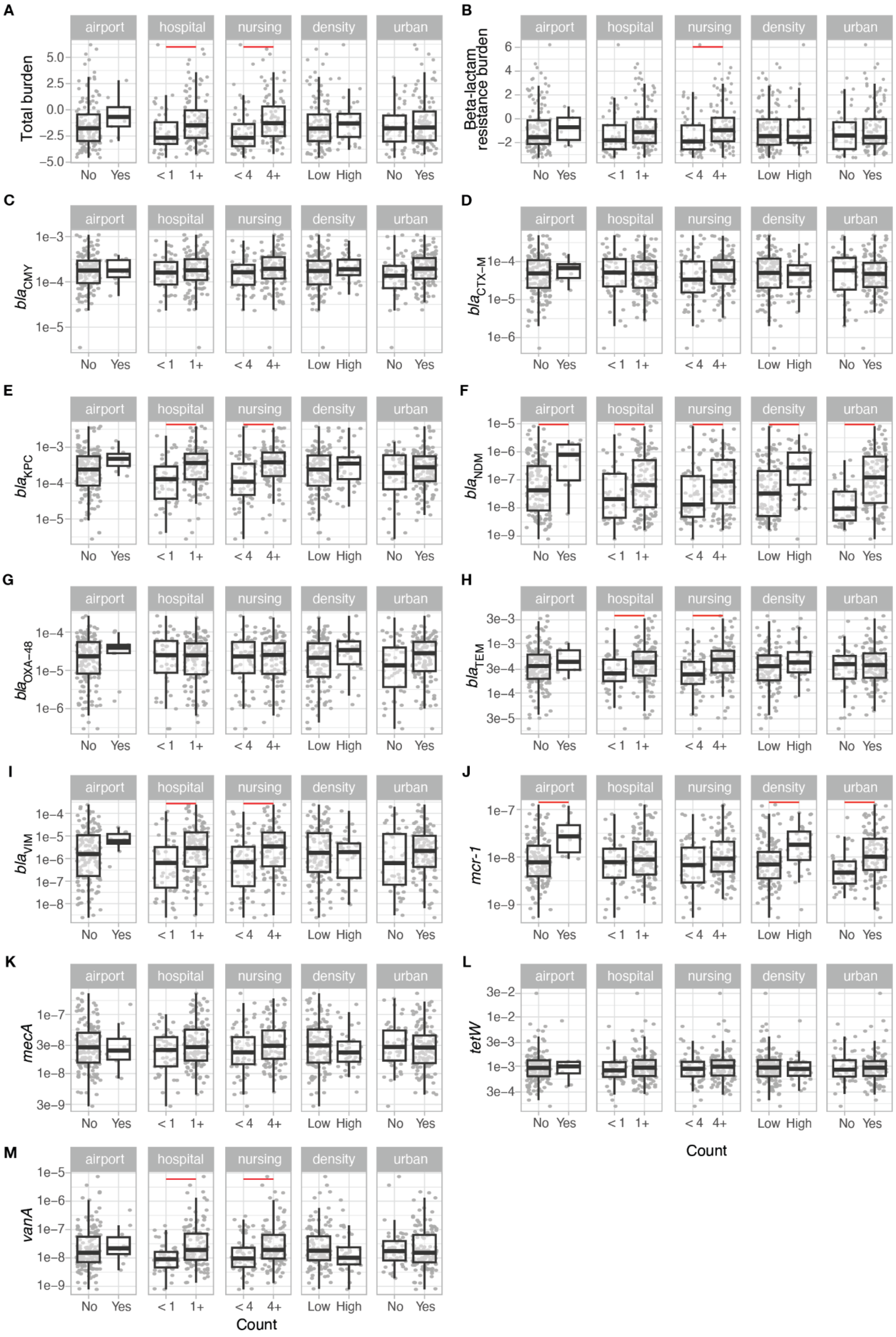

A
B
C
D
E
F
G
H
I
J
K
L
M
airport
hospital
nursing
density
urban
Total burden
Beta-lactam resistance burden
blaCMY
blaCTX−M
blaKPC
blaNDM
blaOXA−48
blaTEM
blaVIM
mcr-1
mecA
tetW
vanA
No
Yes
< 1
1+
< 4
4+
Low
High
Count

Figure S4. Comparison of categorical variables for antibiotic resistance burden and genes. Bivariate analysis of points of interest, population density, and urbanicity for **A)** total resistance burden and **B)** beta-lactamase resistance burden measured in wastewater as aggregate z-scores. Same analysis done for wastewater concentration normalized by 16S rRNA gene for individual genes, **C)** bla_{CMY}, **D)** $bla_{CTX\text{-}M}$, **E)** bla_{KPC}, **F)** bla_{NDM}, **G)** $bla_{OXA\text{-}48}$, **H)** bla_{TEM}, **I)** bla_{VIM}, **J)** *mcr-1*, **K)** *mecA*, **L)** *tetW*, and **M)** *vanA*. The median is shown by the line inside the box with the 25th and 75th percentile represented by the lower and upper boundary of the box. Bottom and top whiskers show 1.5 x interquartile range. Red lines indicate a significant pairwise difference between regions measured by the Wilcoxon post-hoc test with Benjamini-Hochberg correction applied ($p < 0.05$).

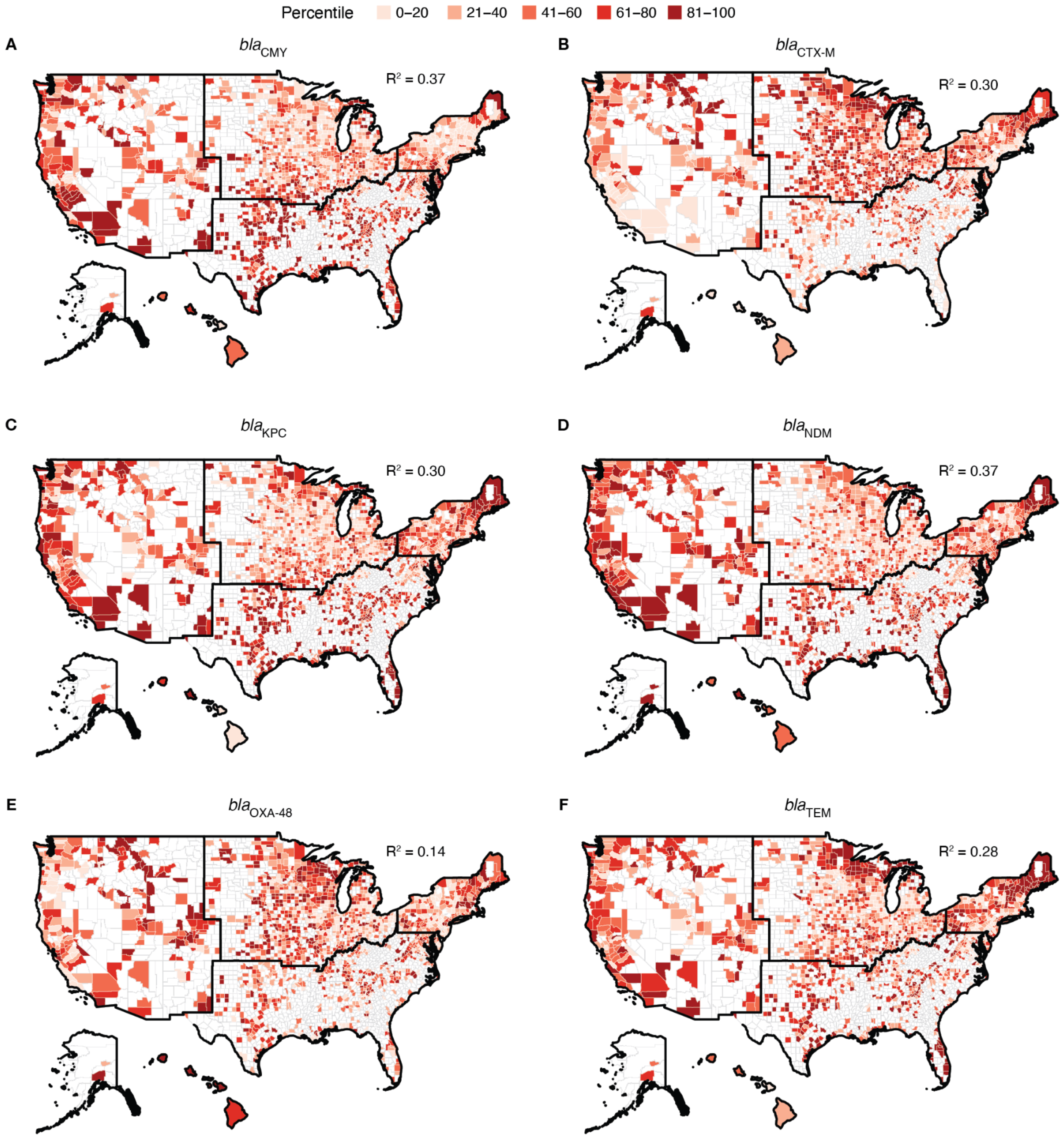
Percentile
0–20
21–40
41–60
61–80
81–100
A
bla_{CMY}
$R^2 = 0.37$
B
bla_{CTX-M}
$R^2 = 0.30$
C
bla_{KPC}
$R^2 = 0.30$
D
bla_{NDM}
$R^2 = 0.37$
E
bla_{OXA-48}
$R^2 = 0.14$
F
bla_{TEM}
$R^2 = 0.28$

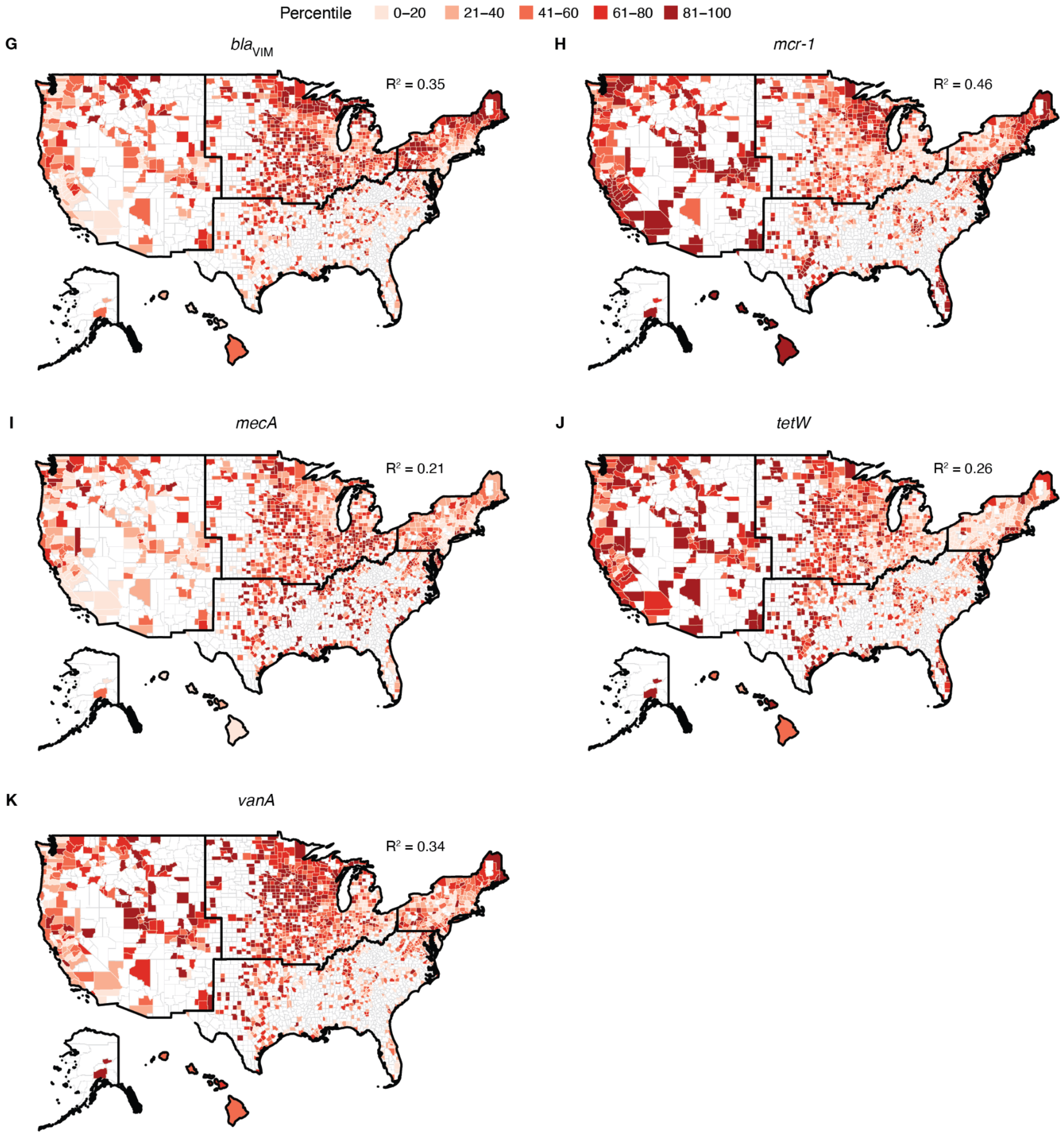

Figure S5. Random forest modeling of antibiotic resistance gene concentrations across the United States using secondary data. Predicted **A)** *bla*$_{CMY}$ (R^2: 0.37), **B)** *bla*$_{CTX\text{-}M}$ (R^2: 0.30), **C)** *bla*$_{KPC}$ (R^2: 0.30), **D)** *bla*$_{NDM}$ (R2: 0.37), **E)** *bla*$_{OXA\text{-}48}$ (R^2: 0.14), **F)** *bla*$_{TEM}$ (R^2: 0.28), **G)** *bla*$_{VIM}$ (R^2: 0.35), **H)** *mcr-1* (R^2: 0.46), **I)** *mecA* (R^2: 0.21), **J)** *tetW* (R^2: 0.26), and **K)** *vanA* (R^2: 0.34) concentrations across the United States. The predicted concentration, normalized by 16S rRNA, is visualized as percentiles, with darker colors indicating higher concentrations. The random forest models are trained based on the secondary data in Table 1 and 2. A higher percentile indicates a higher predicted concentration within that county. Each map shows predictive performance of the model indicated by R^2. Alaska and Hawaii are not to scale.

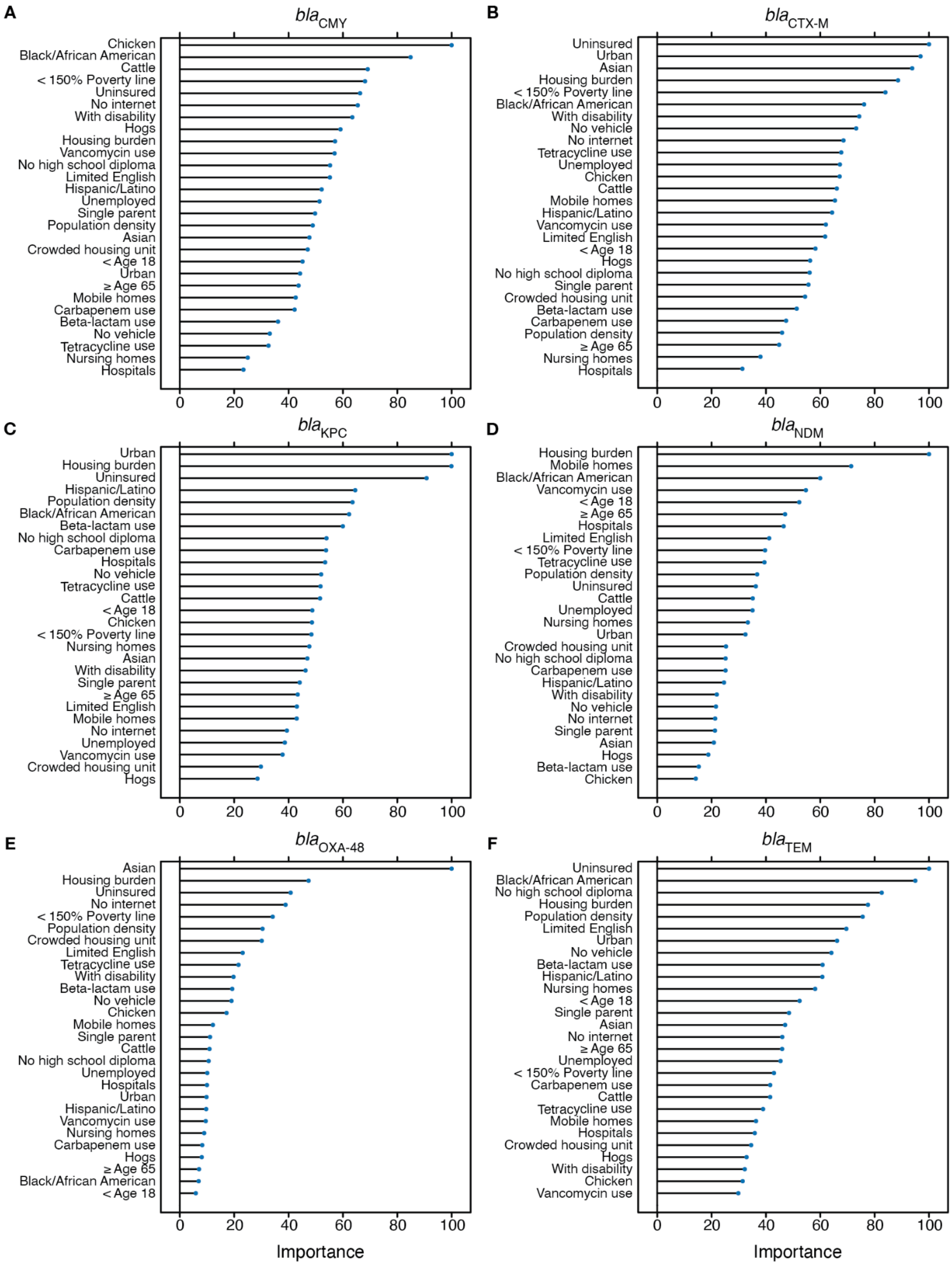

A
bla_{CMY}
Chicken
Black/African American
Cattle
< 150% Poverty line
Uninsured
No internet
With disability
Hogs
Housing burden
Vancomycin use
No high school diploma
Limited English
Hispanic/Latino
Unemployed
Single parent
Population density
Asian
Crowded housing unit
< Age 18
Urban
≥ Age 65
Mobile homes
Carbapenem use
Beta-lactam use
No vehicle
Tetracycline use
Nursing homes
Hospitals
0 20 40 60 80 100
B
bla_{CTX-M}
Uninsured
Urban
Asian
Housing burden
< 150% Poverty line
Black/African American
With disability
No vehicle
No internet
Tetracycline use
Unemployed
Chicken
Cattle
Mobile homes
Hispanic/Latino
Vancomycin use
Limited English
< Age 18
Hogs
No high school diploma
Single parent
Crowded housing unit
Beta-lactam use
Carbapenem use
Population density
≥ Age 65
Nursing homes
Hospitals
0 20 40 60 80 100
C
bla_{KPC}
Urban
Housing burden
Uninsured
Hispanic/Latino
Population density
Black/African American
Beta-lactam use
No high school diploma
Carbapenem use
Hospitals
No vehicle
Tetracycline use
Cattle
< Age 18
Chicken
< 150% Poverty line
Nursing homes
Asian
With disability
Single parent
≥ Age 65
Limited English
Mobile homes
No internet
Unemployed
Vancomycin use
Crowded housing unit
Hogs
0 20 40 60 80 100
D
bla_{NDM}
Housing burden
Mobile homes
Black/African American
Vancomycin use
< Age 18
≥ Age 65
Hospitals
Limited English
< 150% Poverty line
Tetracycline use
Population density
Uninsured
Cattle
Unemployed
Nursing homes
Urban
Crowded housing unit
No high school diploma
Carbapenem use
Hispanic/Latino
With disability
No vehicle
No internet
Single parent
Asian
Hogs
Beta-lactam use
Chicken
0 20 40 60 80 100
E
bla_{OXA-48}
Asian
Housing burden
Uninsured
No internet
< 150% Poverty line
Population density
Crowded housing unit
Limited English
Tetracycline use
With disability
Beta-lactam use
No vehicle
Chicken
Mobile homes
Single parent
Cattle
No high school diploma
Unemployed
Hospitals
Urban
Hispanic/Latino
Vancomycin use
Nursing homes
Carbapenem use
Hogs
≥ Age 65
Black/African American
< Age 18
0 20 40 60 80 100
Importance
F
bla_{TEM}
Uninsured
Black/African American
No high school diploma
Housing burden
Population density
Limited English
Urban
No vehicle
Beta-lactam use
Hispanic/Latino
Nursing homes
< Age 18
Single parent
Asian
No internet
≥ Age 65
Unemployed
< 150% Poverty line
Carbapenem use
Cattle
Tetracycline use
Mobile homes
Hospitals
Crowded housing unit
Hogs
With disability
Chicken
Vancomycin use
0 20 40 60 80 100
Importance

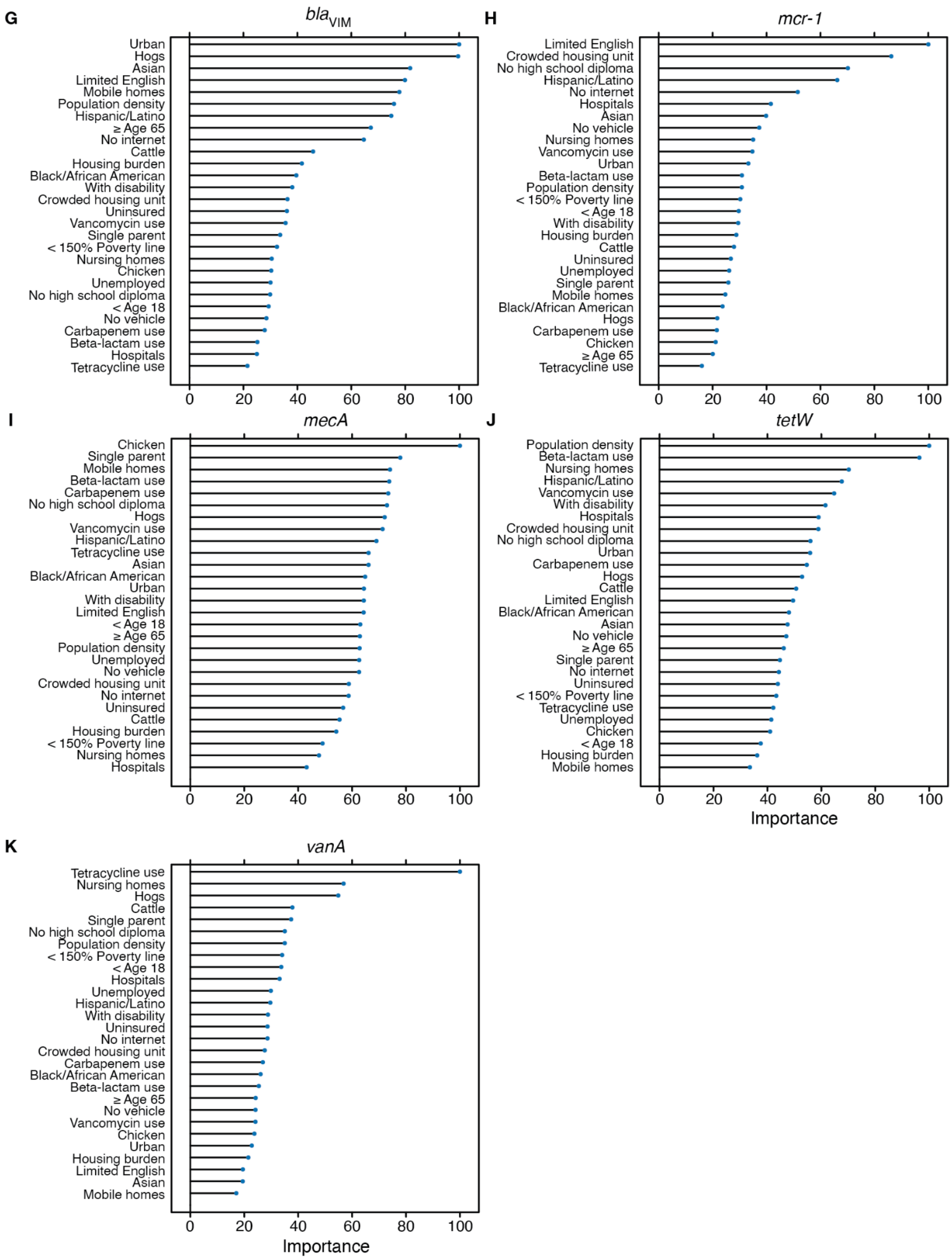

Figure S6. Variable importance for individual ARG random forest prediction model. Random forest model predicts relative concentration of ARGs normalized by 16S rRNA across

all counties in the U.S. for **A)** *bla*$_{CMY}$, **B)** *bla*$_{CTX-M}$, **C)** *bla*$_{KPC}$, **D)** *bla*$_{NDM}$, **E)** *bla*$_{OXA-48}$, **F)** *bla*$_{TEM}$, **G)** *bla*$_{VIM}$, **H)** *mcr-1*, **I)** *mecA*, **J)** *tetW*, and **K)** *vanA*.

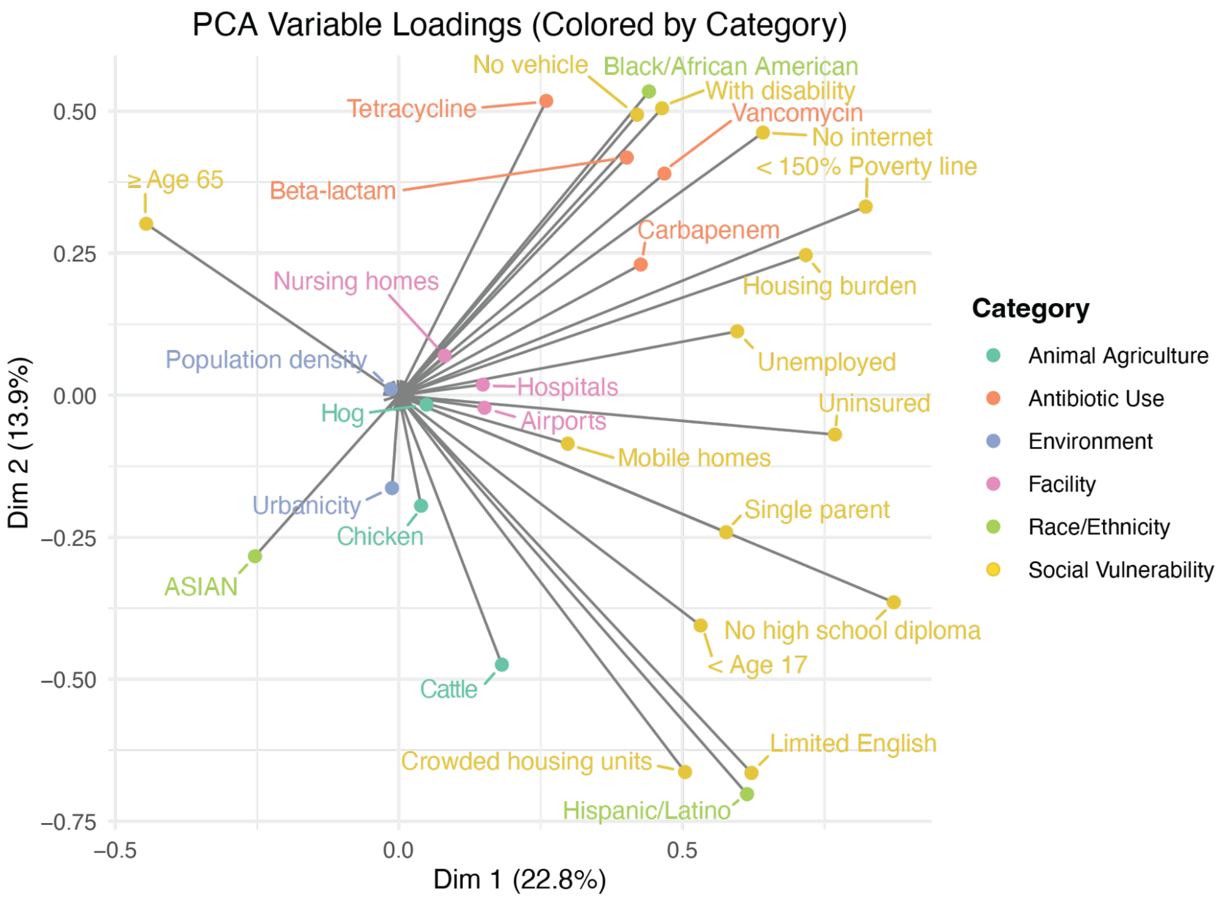

Figure S7. Principle component analysis (PCA) of secondary variables used in the study. A Principal Component Analysis (PCA) was performed on the metadata parameters to investigate the underlying structure and potential co-correlation. Secondary variables are categorized by color. The PCA indicated that the variance within the metadata was distributed across multiple dimensions, with the first two principal components explaining 36.7% of the total variance. Visual inspection of variable loadings revealed that while the social vulnerability indicators generally loaded positively on Dimension 1, suggesting a shared underlying characteristic, they displayed a considerable spread among Dimension 2. Subsequent components (PC3: 11.5%, PC4: 8.7%) captured further, distinct aspects of the data structure.

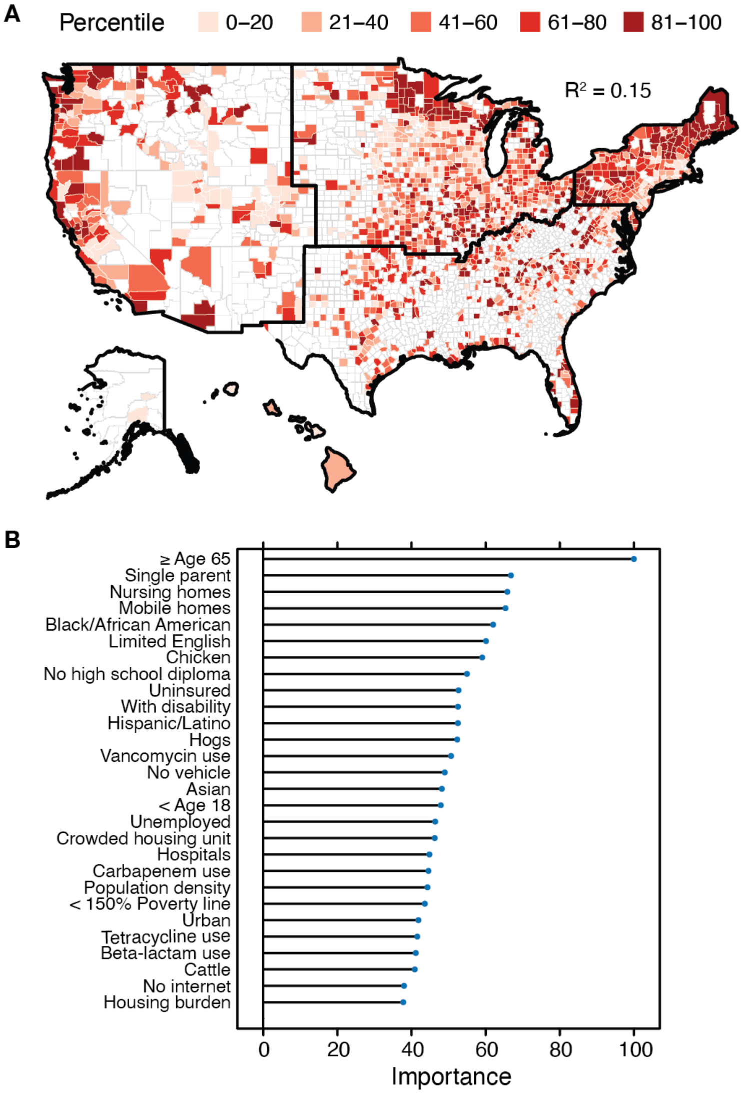

Figure S8. Random forest modeling of overall antibiotic resistance gene prevalence across the United States using secondary data. A) The predicted resistance gene prevalence and **B)** variable importance using cumulative burden z-scores visualized as percentiles, with darker colors indicating higher concentrations. The random forest models are trained based on the secondary data in Table 1 and 2. A higher percentile indicates a higher predicted concentration within that county. Predictive performance of the model indicated by R^2. Alaska and Hawaii are not to scale.

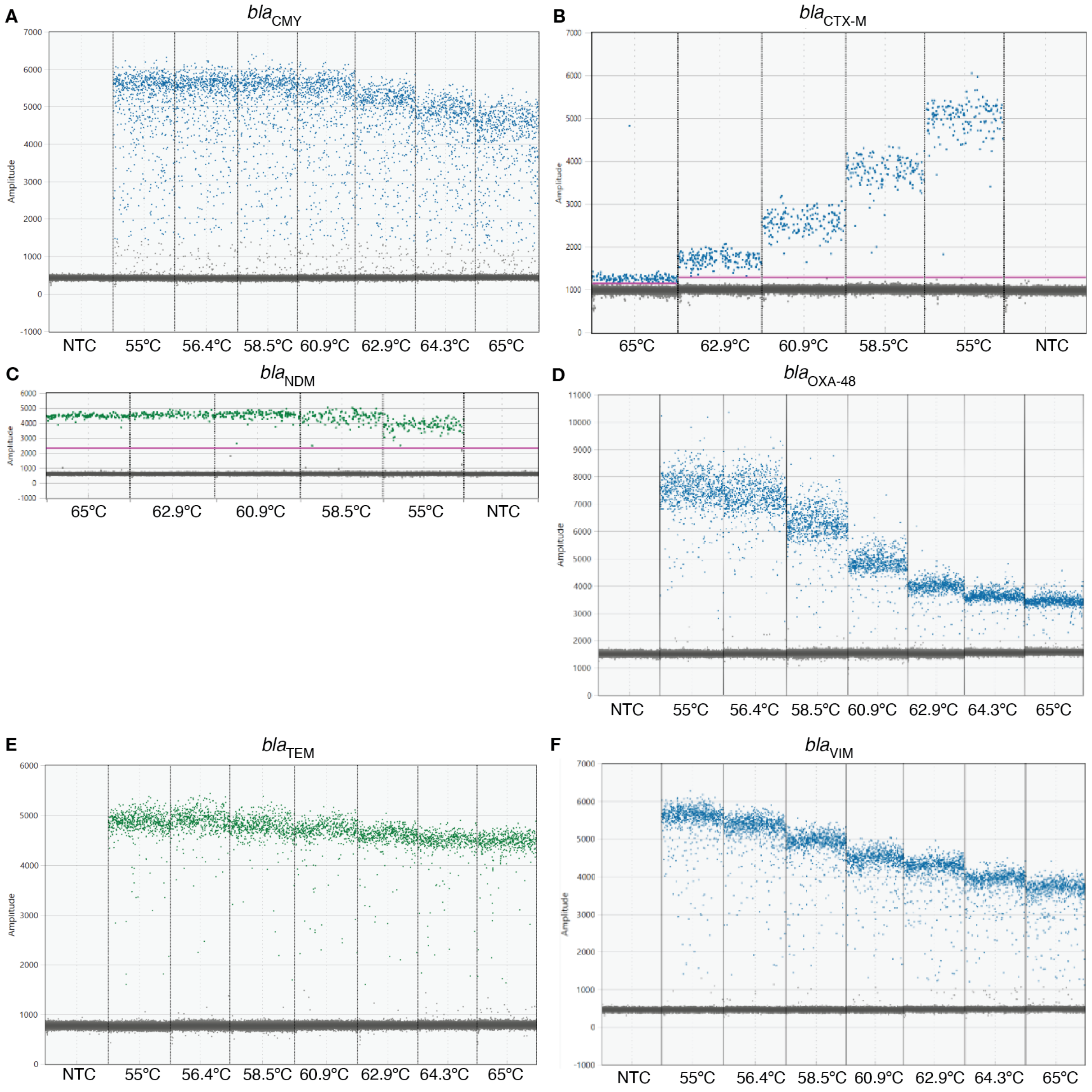

A
blaCMY
Amplitude
NTC
55°C
56.4°C
58.5°C
60.9°C
62.9°C
64.3°C
65°C
B
blaCTX-M
Amplitude
65°C
62.9°C
60.9°C
58.5°C
55°C
NTC
C
blaNDM
Amplitude
65°C
62.9°C
60.9°C
58.5°C
55°C
NTC
D
blaOXA-48
Amplitude
NTC
55°C
56.4°C
58.5°C
60.9°C
62.9°C
64.3°C
65°C
E
blaTEM
Amplitude
NTC
55°C
56.4°C
58.5°C
60.9°C
62.9°C
64.3°C
65°C
F
blaVIM
Amplitude
NTC
55°C
56.4°C
58.5°C
60.9°C
62.9°C
64.3°C
65°C

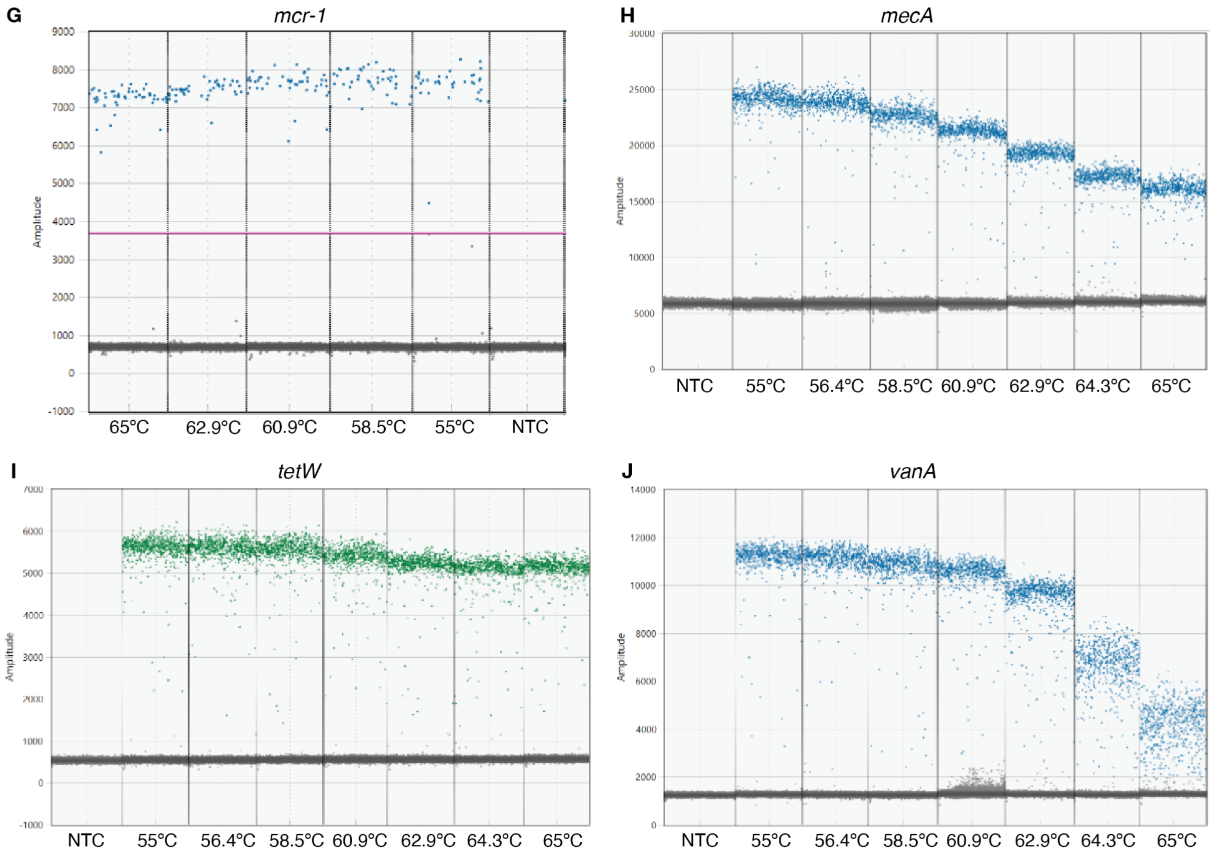

Figure S9. Temperature gradient test results for ARG assays. Screenshots for droplet digital PCR 1D plot of temperature gradient testing for **A)** bla_{CMY}, **B)** $bla_{\text{CTX-M}}$, **C)** bla_{NDM}, **D)** $bla_{\text{OXA-48}}$, **E)** bla_{TEM}, **F)** bla_{VIM}, **G)** *mcr-1*, **H)** *mecA*, **I)** *tetW*, and **J)** *vanA*. Temperature gradient testing was done with gblocks of target sequences (sequences provided in the SI), except for bla_{NDM} and $bla_{\text{CTX-M}}$, which were done with 1:10 and 1:100 diluted wastewater respectively. Probes were either FAM (blue dots in the figure) or HEX (green dots in the figure). The pink line shows the automatic threshold by QX Manager. bla_{KPC} was added later as the last target so instead of a temperature gradient testing, 58°C was tested to confirm that the assay performs well at the temperature set for other assays.

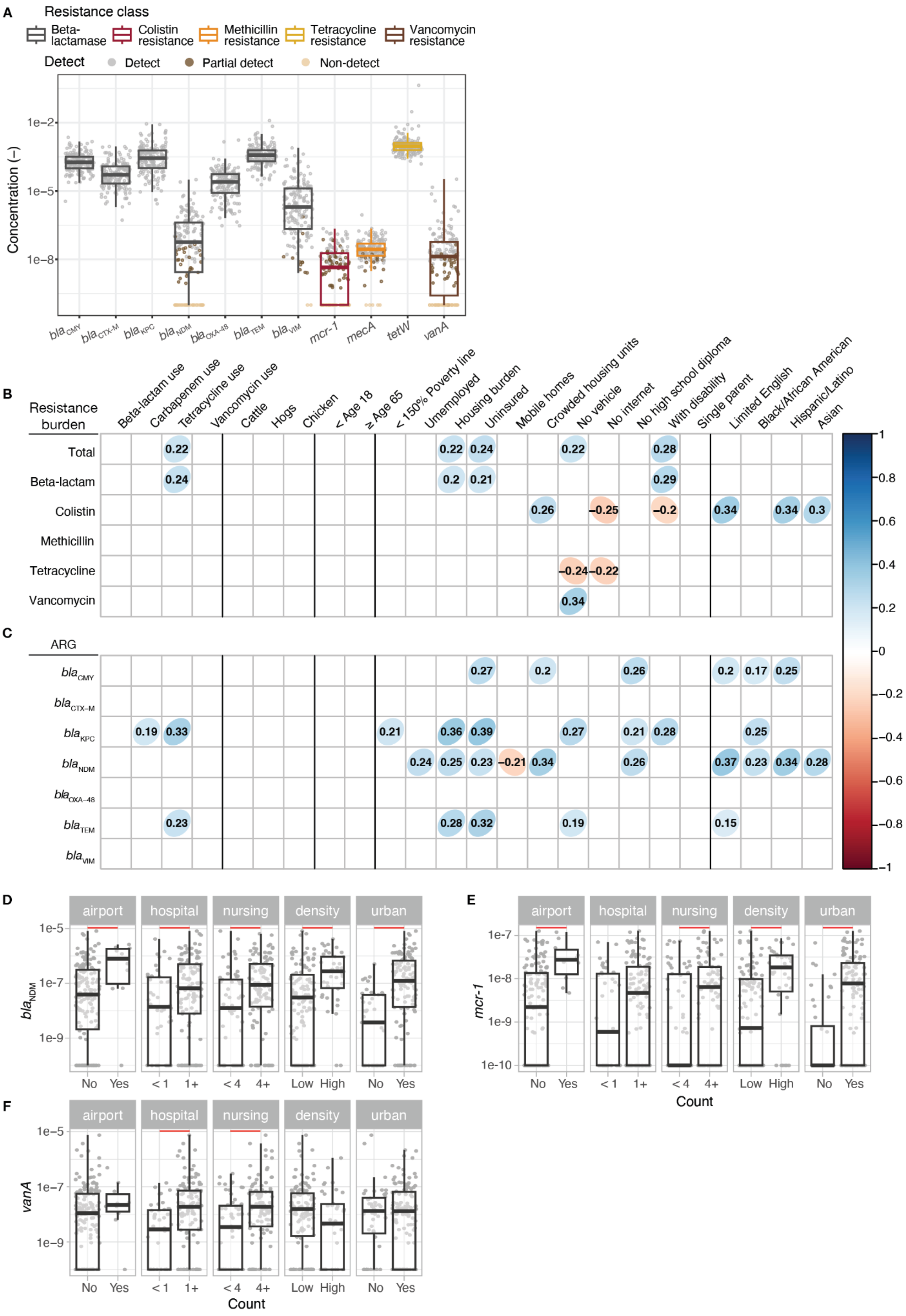
A
Resistance class
Beta-lactamase
Colistin resistance
Methicillin resistance
Tetracycline resistance
Vancomycin resistance
Detect
Detect
Partial detect
Non-detect
Concentration (–)
1e−2
1e−5
1e−8
blaCMY
blaCTX-M
blaKPC
blaNDM
blaOXA-48
blaTEM
blaVIM
mcr-1
mecA
tetW
vanA
B
Resistance burden
Beta-lactam use
Carbapenem use
Tetracycline use
Vancomycin use
Cattle
Hogs
Chicken
< Age 18
≥ Age 65
< 150% Poverty line
Unemployed
Housing burden
Uninsured
Mobile homes
Crowded housing units
No vehicle
No internet
No high school diploma
With disability
Single parent
Limited English
Black/African American
Hispanic/Latino
Asian
Total
Beta-lactam
Colistin
Methicillin
Tetracycline
Vancomycin
C
ARG
D
E
F
airport
hospital
nursing
density
urban
No
Yes
< 1
1+
< 4
4+
Low
High
Count
blaNDM
mcr-1
vanA
1e−5
1e−7
1e−9
1e−10

Fig S10. Analysis using substitution of 0 for ND instead of half theoretical detection limit. **A)** ARG concentration normalized by 16S rRNA gene for all sample sites. **B)** antibiotic resistance burden score and **C)** beta-lactamase gene concentrations measured in wastewater and normalized by 16S rRNA gene ($p < 0.05$ with Benjamin-Hochberg correction applied). Bivariate analysis of points of interest, population density, and urbancity for **D)** bla_{NDM}, **E)** *mcr-1*, and **F)** *vanA* concentration measured in wastewater normalized by 16S rRNA. Each symbol in panels A, D-F represents a wastewater treatment plant. 1e-10 was added to all values to show ND on a log-scale plot. The median is shown by the line inside the box with the 25th and 75th percentile represented by the lower and upper boundary of the box. Bottom and top whiskers show 1.5 x interquartile range. Red lines indicate a significant pairwise difference between regions measured by the Conover-Iman post-hoc test with Benjamini-Hochberg correction applied ($p < 0.05$).

Table S1. **Summary of samples collected per census region.**

	All	Midwest	Northeast	South	West
**# of WWTPs**	163	32	24	48	59
**Total # of samples**	443	92	59	130	162
**Mean # of samples per WWTP**	2.7	2.9	2.5	2.7	2.7

Table S2. Exact p-values for significant pairwise difference ($p < 0.025$) between regions measured by the two-tail Conover-Iman post-hoc test with Benjamini-Hochberg correction applied in Figure 1.

Figure panel	Measurement	Comparison	p-value
C	Total	Midwest-South	0.0207
D	*bla*$_{KPC}$	Midwest-Northeast	0.0018
D	*bla*$_{KPC}$	Midwest-South	0.0000
D	*bla*$_{KPC}$	South-West	0.0005
D	*bla*$_{NDM}$	Midwest-South	0.0005
D	*bla*$_{NDM}$	Midwest-West	0.0004
D	*mcr-1*	Midwest-South	0.0022
D	*mcr-1*	Midwest-West	0.0000
D	*mcr-1*	Northeast-South	0.0202
D	*mcr-1*	Northeast-West	0.0001
D	*mcr-1*	South-West	0.0129
D	*vanA*	Midwest-West	0.0244

Table S3. Statistics for Wilcoxon post-hoc test in Figure 2.

	Test statistics	Confidence interval	Effect size	Degrees of freedom	Adjusted p-values
Total burden - hospital	1432	(-1.6, -0.27)	0.23	142	0.017
Total burden - nursing	1503	(-1.8, -0.61)	0.32	142	0.0007
Colistin burden - airport	272	(-0.84, -0.17)	0.27	155	0.002
Colistin burden - density	1204	(-0.43, -0.10)	0.31	155	2.6e-4
Colistin burden - urban	1352	(-0.23, -0.08)	0.35	155	6.0e-5
bla_{NDM} - airport	354	(-2.6e-6, 8.1e-6)	0.22	160	0.005
bla_{NDM} - hospital	1969	(-8.1e-6, 4.1e-6)	0.18	160	0.022
bla_{NDM} - nursing	2096	(-6.4e-6, 8.1e-6)	0.25	160	0.001
bla_{NDM} - density	1329	(-4.1e-6, 8.1e-6)	0.32	160	6.1e-5
bla_{NDM} - urban	1056	(-8.1e-6, 5.0e-7)	0.45	160	1.5e-8

Table S4. Mean and standard deviation of the total number of copies of target per reaction for each target

Target	n	mean	sd
bla_{CMY}	445	0.0451	0.0607
bla_{CTX-M}	445	0.0175	0.0328
bla_{KPC}	445	0.0766	0.118
mecA	444	0.000735	0.00112
bla_{NDM}	445	0.0106	0.05
bla_{OXA-48}	442	0.00733	0.0184
bla_{TEM}	445	0.0844	0.102
bla_{VIM}	445	0.128	0.214
mcr-1	442	0.000260	0.000474
tetW	445	0.183	0.169
vanA	445	0.00866	0.072
16S rRNA	439	0.321	0.215

Copies per target calculated by dividing the number of positive droplets by the number of accepted droplets. Note that mean and sd were calculated using all samples, not just those with detectable target.

Preparation of secondary data

Demographic and socioeconomic factors. The 2022 Social Vulnerability Index (SVI) from the US CDC includes various demographic and socioeconomic factors from the 2022 5-year American Community Survey (ACS) that relate to a community's social vulnerability or ability to respond to external hazards.[12] For each sewershed, we approximated the value of selected variables used in the SVI as a proportion using the relevant ACS variables obtained at the census tract resolution (**Table S5**).[13] Census tract and sewershed boundaries did not align, so we first aggregated ACS variable counts across all census tracts intersecting a sewershed to estimate counts for each sewershed. Specifically, we used the Tabulate Intersection geoprocessing tool in ArcGIS Pro (version 3.1.1) to determine the area proportion *p* of each intersecting census tract *n* in the sewershed.[14] Next, we adjusted the census tract-level count of each ACS variable based on *p* (**Equation 1**). Then we summed the adjusted counts across all *N* census tracts intersecting the sewershed to determine the sewershed-level count for each ACS variable (**Equation 2**). Using sewershed-level counts of each ACS variable, we calculated the sewershed-level value of each SVI variable as a proportion as described in **Table S5**. The distribution of each proportion among sewersheds in the study is shown in **Figure S11**.

Equation 1. $\text{count\_adjusted}_n = \text{count}_n \times p_n$

Equation 2. $\text{count\_sewershed} = \sum_{n=1}^{N} \text{count\_adjusted}_n$

Table S5. Selected main and adjunct variables in the Social Vulnerability Index (SVI) calculated as proportions using relevant American Community Survey (ACS) variables

Proportion	Description	Calculation using ACS variables[a]
< 150% Poverty line	Proportion of the population for whom poverty status is determined below the 150% poverty line	S1701_C01_040E / S1701_C01_001E
Unemployed	Proportion of the civilian labor force age 16+ years that is unemployed	DP03_0005E / DP03_0003E
Housing burden	Proportion of occupied housing units that are housing cost-burdened with an annual income <$75K	(S2503_C01_028E + S2503_C01_032E + S2503_C01_036E + S2503_C01_040E) / S2503_C01_001E
No high school diploma	Proportion the population age 25+ years with no high school diploma	B06009_002E / B06009_001E
Uninsured	Proportion of the civilian noninstitutionalized population that is uninsured	S2701_C04_001E / S2701_C01_001E
≥ Age 65	Proportion of the population age 65+ years	S0101_C01_030E / S0601_C01_001E
< Age 18	Proportion of the population age 17 years and younger	DP05_0019E / S0601_C01_001E

With disability	Proportion of the civilian noninstitutionalized population with a disability	DP02_0072E / S2701_C01_001E
Single parent	Proportion of single-parent households with children <18 years	(DP02_0007E + DP02_0011E) / (DP02_0006E + DP02_0010E)
Limited English	Proportion of persons age 5+ years who speak English "less than well"	(B16005_007E + B16005_008E + B16005_012E + B16005_013E + B16005_017E + B16005_018E + B16005_022E + B16005_023E + B16005_029E + B16005_030E + B16005_034E + B16005_035E + B16005_039E + B16005_040E + B16005_044E + B16005_045E) / B16005_001E
Black/African American	Proportion of the population that is Black/African American, not Hispanic or Latino	DP05_0080E / S0601_C01_001E
Hispanic/Latino	Proportion of the population this is Hispanic or Latino	DP05_0073E / S0601_C01_001E
Asian	Proportion of the population that is Asian, not Hispanic or Latino	DP05_0082E / S0601_C01_001E
Mobile homes	Proportion of housing units that are mobile homes	DP04_0014E / DP04_0001E
Crowded housing units	Proportion of occupied housing units with more people than rooms	(DP04_0078E + DP04_0079E) / DP04_0002E
No vehicle	Proportion of occupied housing units with no vehicle	DP04_0058E / DP04_0002E
No internet	Proportion of households with no internet	S2801_C01_019E / DP02_0001E

[a] Variables are from the 2022 5-year American Community Survey (ACS). Census tract-level counts for each variable were first adjusted and summed by sewershed to obtain sewershed-level counts.

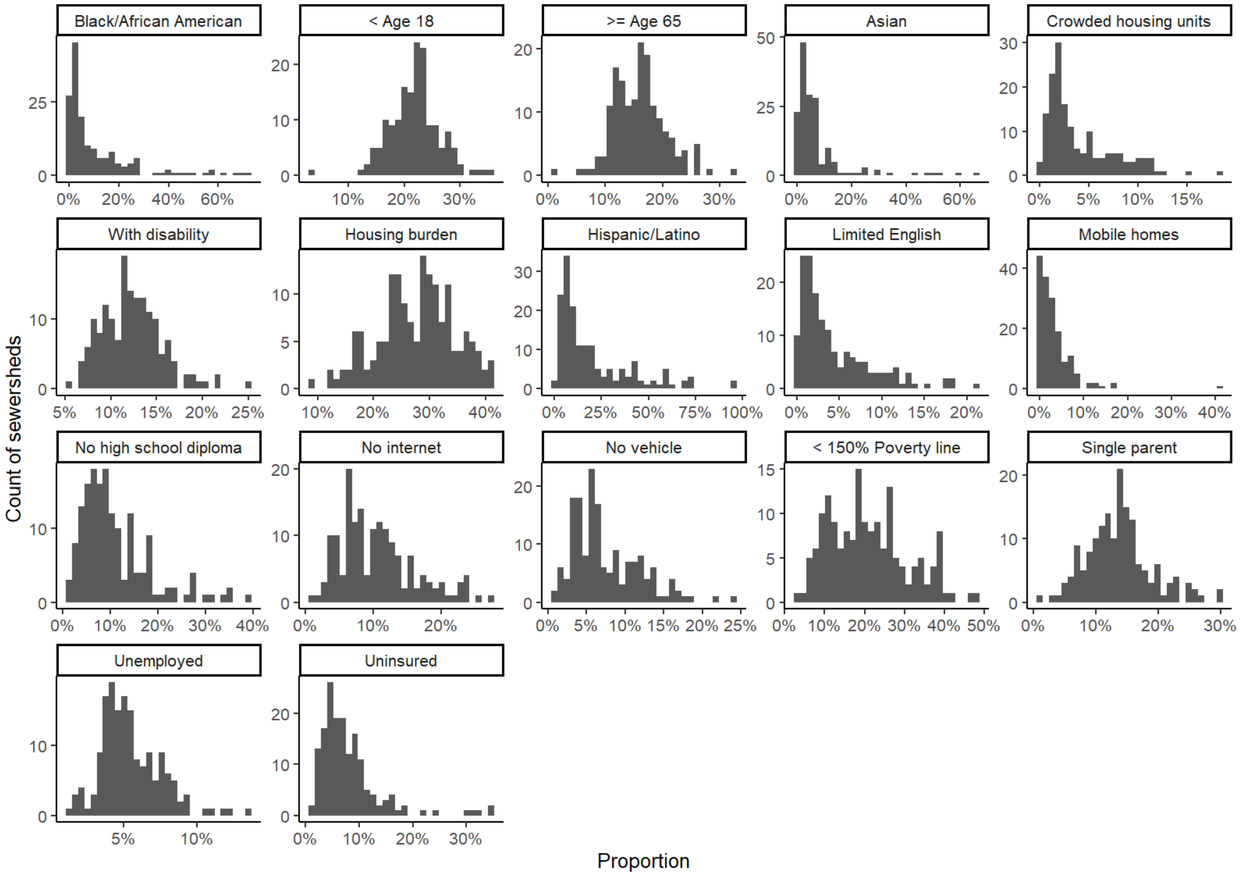

Figure S11. **Distribution of each variable in Table S5 among sewersheds.**

Urbanicity and population density. We used 2020 urban-rural designations from the US Census Bureau to determine the extent to which sewersheds are classified as urban.[15] For each sewershed, we used the Tabulate Intersection geoprocessing tool in ArcGIS Pro to determine the area proportion of the sewershed that intersects any urban area. We assigned a value of 0 if a sewershed did not intersect any urban area. The distribution of the urban proportion among sewersheds in this study is shown in **Figure S12**. Sewersheds with an urban area proportion ≥ 50% were classified as urban and sewersheds with an urban area proportion < 50% were classified as non-urban for data analysis. To determine population density, we obtained the population of each county from the 2022 5-year ACS (subject table S0101) and the land area in square meters of each county from the 2022 US Census Bureau county boundaries (5-meter resolution).[13,14] We calculated the population density in square kilometers of each county by dividing the population by the land area and multiplying by 1000^2. We assigned the population density of each sewershed as the population density of its predominant county (i.e., the county that the majority of the sewershed is located in). We determined the predominant county of each sewershed using the Tabulate Intersection geoprocessing tool in ArcGIS Pro and 2022 county boundaries (5-meter resolution) from the US Census Bureau.[14] The distribution of population

density among sewersheds in this study is shown in **Figure S13**. Population density was stratified by 617 per km^2, the average number of people in cities in the U.S., for data analysis.

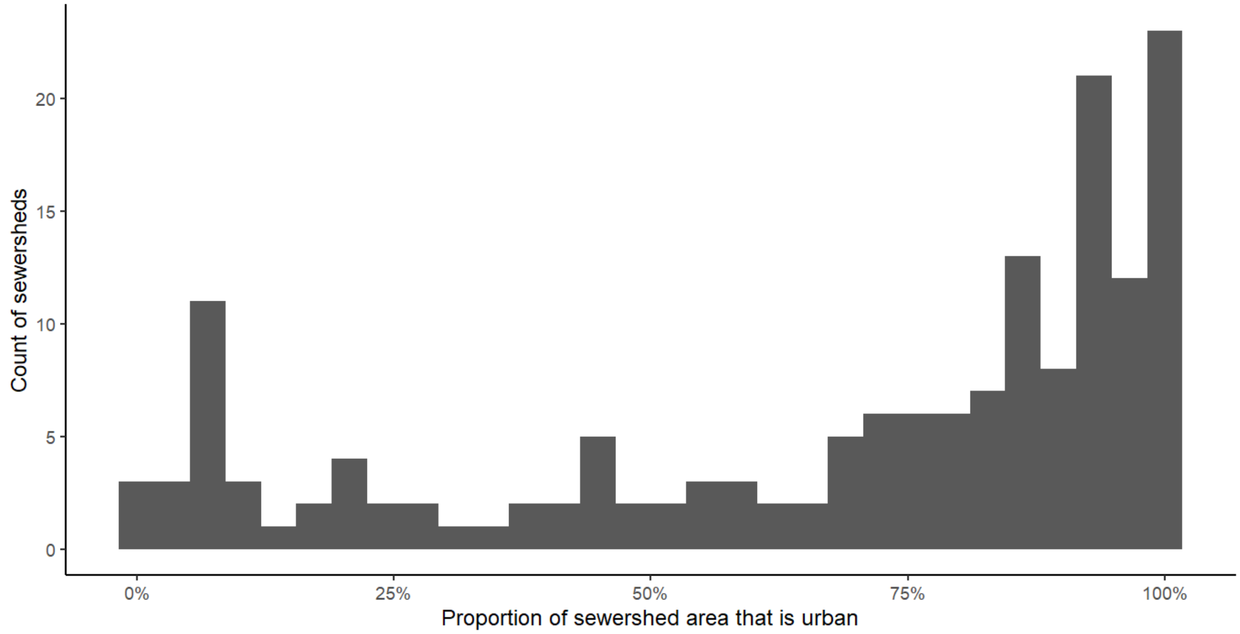

Figure S12. **Distribution of urbanicity among sewersheds.**

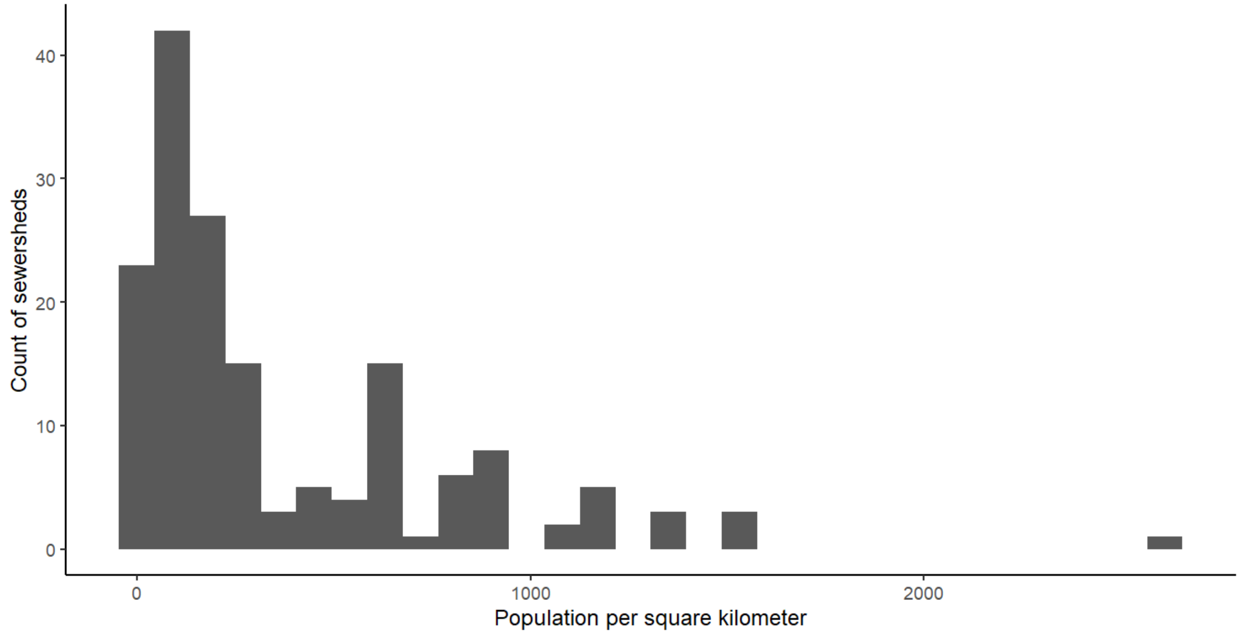

Figure S13. **Distribution of population density among sewersheds.**

Points of interest. We obtained the locations of major airports (defined as usage category "1,000,000 or more") from Esri (source: Federal Aviation Administration's National Airspace System Resource Aeronautical Data Product).[16] We obtained the locations of hospitals and nursing homes from the US Department of Homeland Security's Homeland Infrastructure Foundation-Level Data database.[17] We used the Tabulate Intersection geoprocessing tool in

ArcGIS Pro to determine the number of airports, hospitals, and nursing homes in each sewershed. For hospitals and nursing homes, we omitted any locations with a non-open status prior to using the Tabulate Intersection tool. **Figure S14** shows the distribution of airports, hospitals, and nursing homes among sewersheds in this study. We could not weight hospitals and nursing homes by size due to not all sites reporting a valid value for population or beds. For data analysis, airports and hospitals were split into groups based on the presence of the facilities, and the number of nursing homes were split by the national median number of nursing homes (median: 4).

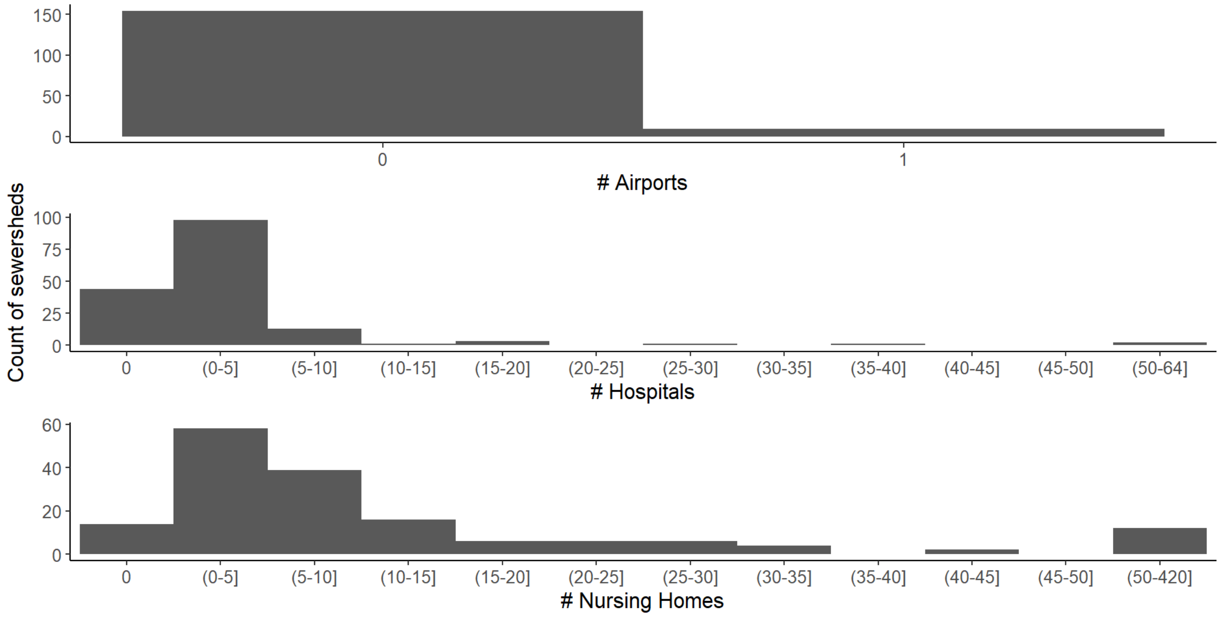

Figure S14. **Distribution of points of interest among sewersheds.**

Agricultural activity. Using the US Department of Agriculture (USDA) National Agricultural Statistics Service (NASS), we obtained 2022 inventory numbers for cattle, chickens, and hogs at the county resolution (**Table S6**).[18] For each sewershed, we assigned the inventory numbers of its predominant county (described above) to use as an indicator of agricultural activity in the sewershed. If inventory values were not reported or withheld to disclose numbers for individual operations, we assumed a value of zero. The Newark, NJ sewershed had no reported data for its predominant county, so we assigned inventory numbers from its second most predominant county to use for the analysis. The Census Bureau provides boundaries for county equivalents in Connecticut rather than counties which are used in the USDA NASS dataset. We assumed the Western Planning Region county equivalent is most similar to Fairfield county to determine the predominant county for the Stamford, CT sewershed. For chickens, we summed over all data items (broilers, layers, pullets, roosters). The distribution of cattle, chickens, and hogs among sewersheds in the study is shown in **Figure S15**.

Table S6. US Department of Agriculture National Agricultural Statistics Service Selections

**Program**	**Census**
Sector	Animals & Products
Group	Poultry; Livestock
Commodity	Cattle; Chickens; Hogs
Category	Inventory
Data Item	Cattle, Incl Calves - Inventory<br>Chickens, Broilers - Inventory<br>Chickens, Layers - Inventory<br>Chickens, Pullets, Replacement - Inventory<br>Chickens, Roosters - Inventory<br>Hogs - Inventory
Domain	Total
Geographic Level	County
State	All
Year	2022
Period Type	Point in Time
Period	End of Dec

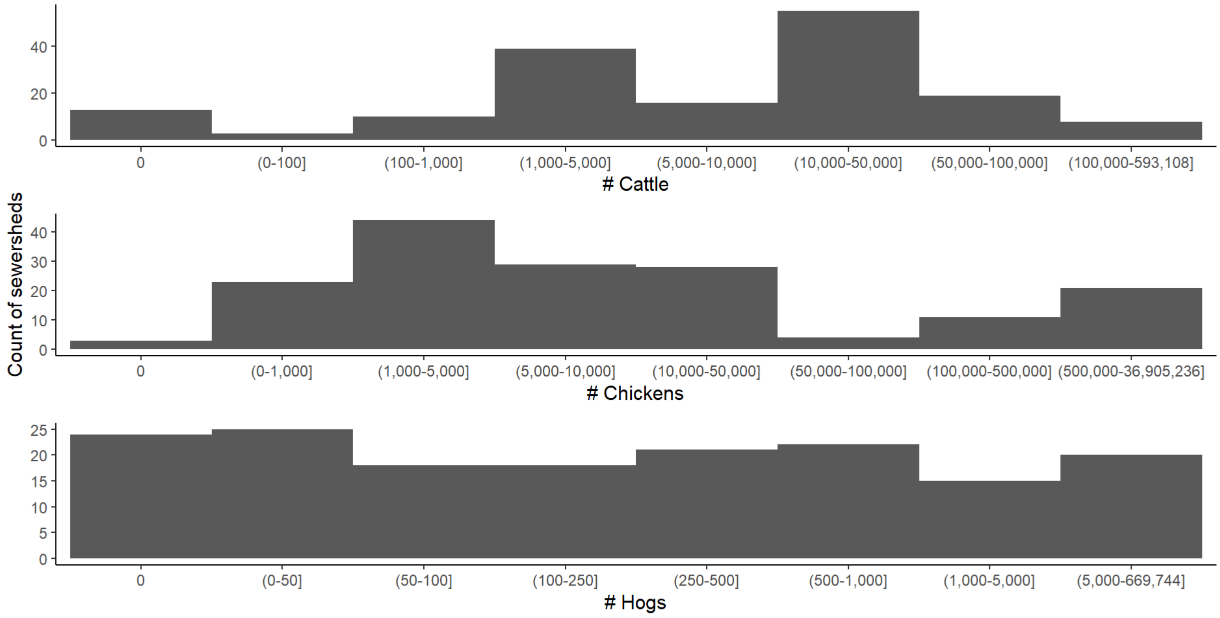

Figures S15. Distribution of agriculture animal inventory numbers among sewersheds.

Epic Cosmos data. All encounters in the Epic Cosmos dataset were temporally limited to encounters from June 1, 2023–June 1, 2024. Encounters for sampled sewersheds were further geographically limited to the 116 counties representing the predominant county serviced by wastewater treatment plants (described above). Encounters were then filtered for all encounters with beta-lactam antibiotics (penicillins, cephalosporins, and carbapenems), tetracycline, vancomycin, and colistin antibiotics dispensed. The annual numbers of encounters with these prescriptions were then divided by the total number of encounters in the analysis period for each county. Minimal colistin use was reported in the Epic dataset, so this variable was ultimately not included.

Additional details on modeling

Feature set. Our prediction model involved minor, strategic adjustments due to data limitations and availability. Adjustments made included: 1) sparse features, such as airport presence, were removed to mitigate the risk of overfitting to rare observations. 2) Conversely, the national antibiotic usage data became available during the project and was included to represent an important mechanistic driver of ARG prevalence. 3) to prevent extensive extrapolation to unsewered areas, the model's predictive scope was limited to U.S. counties where secondary data values fell within the minimum and maximum ranges of the training set for all variables. The impact of these feature changes on model performance is detailed in Table S7. While R^2 values for individual ARGs remained largely consistent with minor fluctuations, the R^2 or the overall summed ARG metric increased substantially, rising from 0.02 to 0.15. However, even with this increase, the total resistance metric was not able to explain as much variance as the predictive models for individual genes, reinforcing the idea that focusing on provides more insightful detail than relying on a summary metric of antibiotic resistance when there are multiple classes of antibiotics and different mechanisms of resistance involved.

Despite being mechanistically relevant, the antibiotic usage variables did not rank highly among the overall important predictors in the final random forest models (Figure 4). This observation is consistent with our correlational analysis, which demonstrated that certain socioeconomic and demographic factors exhibited stronger associations with ARG concentrations in wastewater than the available antibiotic usage metrics. The model's low prioritization of usage variables reinforces the conclusion that broader societal contexts play a key role in ARG prevalence.

Table S7. Model performance changes with adjustment to feature set.

Prediction model target	Initial R^2	Adjusted feature set R^2
bla_{CMY}	0.41	0.37
$bla_{CTX\text{-}M}$	0.29	0.30
bla_{KPC}	0.30	0.30
bla_{NDM}	0.31	0.37
$bla_{OXA\text{-}48}$	0.25	0.14

bla$_{TEM}$	0.26	0.28
bla$_{VIM}$	0.30	0.35
mcr-1	0.40	0.46
mecA	0.22	0.21
tetW	0.29	0.26
vanA	0.36	0.34
Total resistance	0.02	0.15

Sensitivity analysis. To validate our choice of selecting one random sewershed when a county was represented more than once in the dataset, we performed a sensitivity analysis using 50 different independent trials of random selections for each target gene. Overall, the model exhibited tightly clustered predictive performance across the majority of the target genes (**Table S8, Figure S16**). The model's predictive power, measured by the mean R2, clusters around 0.26 to 0.33 for most genes with the highest average R2 score achieved for *mcr-1* (0.39) and the lowest score for *bla*$_{OXA-48}$ (0.18). The repeated trials did not lead to large swings in performance as shown by the tight clustering for each gene. The high stability for most targets confirms that this modest performance is a consistent limitation of the feature set, rather than a matter of noisy training. Therefore the features used in the model are likely capturing relevant information, but additional features are required to significantly increase the overall predictive accuracy, suggesting presence of additional drivers of antibiotic resistance that were not captured in this study and highlighting the complexity of factors that contribute to antibiotic resistance.

Table S8. Model performance stability across different randomly selected sewershed for counties represented by multiple sewersheds

Target	R^2 mean ± standard deviation	R^2 range	Number of trials
bla$_{CMY}$	0.28 ± 0.057	[0.181, 0.443]	50
bla$_{CTX-M}$	0.27 ± 0.032	[0.224, 0.355]	50
bla$_{KPC}$	0.271 ± 0.042	[0.189, 0.375]	50
bla$_{NDM}$	0.329 ± 0.043	[0.266, 0.476]	50
bla$_{OXA-48}$	0.182 ± 0.052	[0.091, 0.343]	50

*bla*TEM	0.26 ± 0.046	[0.17, 0.366]	50
*bla*VIM	0.334 ± 0.046	[0.238, 0.454]	50
mcr-1	0.39 ± 0.061	[0.262, 0.498]	50
mecA	0.261 ± 0.04	[0.186, 0.331]	50
tetW	0.26 ± 0.039	[0.201, 0.4]	50
vanA	0.317 ± 0.026	[0.259, 0.392]	50

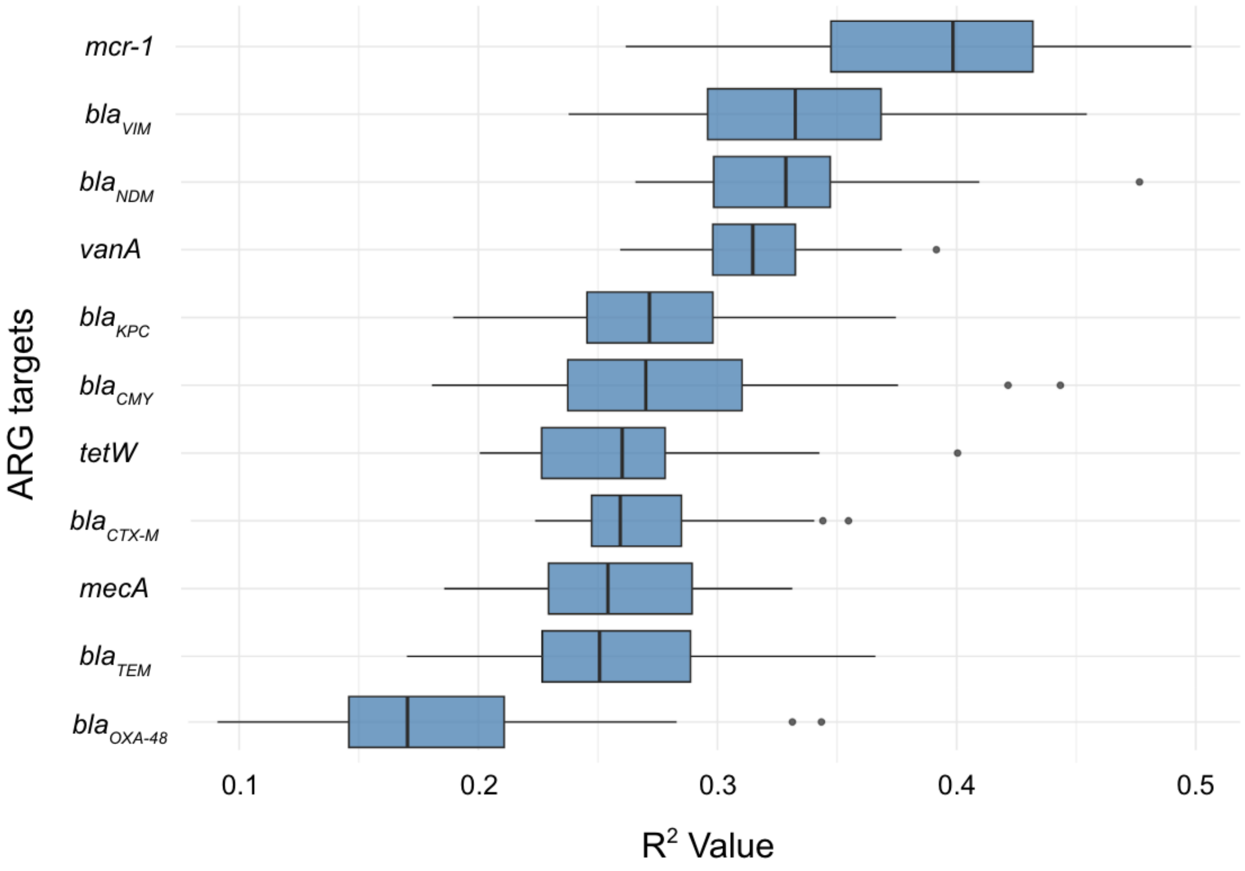

Figure S16. Model performance stability across different randomly selected sewershed for counties represented by multiple sewersheds. The median is shown by the line inside the box with the 25th and 75th percentile represented by the lower and upper boundary of the box. Bottom and top whiskers show 1.5 x interquartile range.

Environmental Microbiology Minimum Information Checklist

Study Description

Study: [Study Name]
Date: 07-Jul-2021
Completed by: [Filled By...]

Environmental Sampling	Sample Treatment	Sample Reduction	Nucleic Acid Extraction	Reverse Transcription	PCR Detection	Analysis
Described in methods section	☐ Performed<br>No sample treatment performed	☐ Performed<br>Centrifugation was used, as described in the methods and referenced paper	Methods described briefly in pre-analytical processing and nucleic-acid extraction section and in references.	☐ Performed<br>Not performed	☐ qPCR ■ dPCR<br>Methods provided including the dMIQE checklist	QA/QC criteria and analysis described in methods

Control Checklist

	Environmental Sampling	Sample Treatment	Sample Reduction	Nucleic Acid Extraction	Reverse Transcription	PCR Detection
Step performed	■	☐	■	■	☐	■
**Negative Controls**						
Step has control info	☐	☐	☐	☐	☐	■
# control replicates	0	0	0	3	0	3
Control result reported	☐	☐	☐	☐	☐	■
Data handling reported	☐	☐	☐	☐	☐	■
**Positive Controls**						
Control introduced	☐	☐	☐	■	☐	■
Internal/External	N/A	N/A	Internal	External	N/A	External
Independent/Parallel	N/A	N/A	Parallel	Independent	N/A	Independent
Step has control info	☐	☐	☐	■	☐	■
# control replicates	0	0	0	1	0	1
Control result reported	☐	☐	☐	■	☐	■
Data Handling reported	☐	☐	☐	■	☐	■

Process Checklist

Environmental Sampling
- ■ Sampling Procedure
- ■ Number of samples
- ■ Sample amount, mean, range
- ■ Sampling locations, dates, times

Sample Treatment
- ☐ Performed
- ☐ Treatment procedure
- ☐ Reagents

Sample Reduction
- ■ Performed
- ■ Reduction procedure
- ■ Reagents
- ☐ Concentration Factor

Nucleic Acid Extraction
- ■ Extraction procedure
- ■ Amount extracted, amount obtained
- ■ Extract storage conditions

qPCR or dPCR
- ■ Target gene name, amplicon length
- ■ Thermocycling temperatures and times
- ■ Master mix: composition, vendors, concentrations
- ■ Additives: vendors, concentrations
- ■ Template amount added, pre-treatment (if any)
- ■ Primers: sequences, concentrations, vendors, references
- ■ Amplicon confirmation method (probe, melt curve, etc)
- ■ Probe sequence, concentration, vendor, reference
- ■ Instrumentation
- ☐ Equivalent volume of sample analyzed by PCR
- ■ Inhibition assessment procedure
- ■ Inhibition control description (if used)
- ■ Number samples tested and found inhibited

Reverse Transcription
- ☐ Performed
- ☐ One or two step
- ☐ cDNA storage conditions (if two step)
- ☐ Reaction temperatures and times
- ☐ Reaction reagents and concentrations
- ☐ Priming method
- ☐ Reaction volume, added template amount
- ☐ Inhibition assessment procedure
- ☐ Inhibition control description (if used)
- ☐ Number samples tested and found inhibited

Analysis – dPCR
- ■ Threshold settings
- ■ Technical replicates, number, well merging
- ■ Partitions measured, number, mean, variance
- ■ Partition volume
- ■ Target copies per partition, mean, variance
- ■ Program used for dPCR analysis
- ■ Explanation of control results, example plots

Analysis – qPCR
- ☐ Method for handling failed negative controls
- ☐ Technical replicates, number, calculations
- ☐ Calibration standards: description and source
- ☐ Method of quantifying standards
- ☐ Calibration curve slope
- ☐ Calibration curve R2
- ☐ Lowest standard measured or 95% LOD
- ☐ Cq value determination method

References

1. Schmidt, G. V. *et al.* Sampling and Pooling Methods for Capturing Herd Level Antibiotic Resistance in Swine Feces using qPCR and CFU Approaches. *PLOS ONE* **10**, e0131672 (2015).
2. Pholwat, S. *et al.* Genotypic antimicrobial resistance assays for use on E. coli isolates and stool specimens. *PLOS ONE* **14**, e0216747 (2019).
3. Garcia, L. S. *Clinical Microbiology Procedures Handbook, 3rd Ed.* (ASM Press, 2010).
4. Böckelmann, U. *et al.* Quantitative PCR Monitoring of Antibiotic Resistance Genes and Bacterial Pathogens in Three European Artificial Groundwater Recharge Systems. *Appl. Environ. Microbiol.* **75**, 154–163 (2009).
5. Chavda, K. D. *et al.* Evaluation of a Multiplex PCR Assay To Rapidly Detect Enterobacteriaceae with a Broad Range of β-Lactamases Directly from Perianal Swabs. *Antimicrob. Agents Chemother.* **60**, 6957–6961 (2016).
6. Lutgring, J. D. *et al.* Phenotypic and Genotypic Characterization of *Enterobacteriaceae* Producing Oxacillinase-48–Like Carbapenemases, United States. *Emerg. Infect. Dis.* **24**, 700–709 (2018).
7. Roschanski, N., Fischer, J., Guerra, B. & Roesler, U. Development of a Multiplex Real-Time PCR for the Rapid Detection of the Predominant Beta-Lactamase Genes CTX-M, SHV, TEM and CIT-Type AmpCs in Enterobacteriaceae. *PLoS ONE* **9**, e100956 (2014).
8. Yang, D. *et al.* The Occurrence of the Colistin Resistance Gene mcr-1 in the Haihe River (China). *Int. J. Environ. Res. Public. Health* **14**, 576 (2017).
9. Smith, M. S. *et al.* Quantification of Tetracycline Resistance Genes in Feedlot Lagoons by Real-Time PCR. *Appl. Environ. Microbiol.* **70**, 7372–7377 (2004).
10. Farivar, T. N. *et al.* Development and evaluation of a Quadruplex Taq Man real-time PCR assay for simultaneous detection of clinical isolates of Enterococcus faecalis, Enterococcus

faecium and their vanA and vanB genotypes. *Iran. J. Microbiol.* **6**, 335–340 (2014).

11. Suzuki, M. T., Taylor, L. T. & DeLong, E. F. Quantitative Analysis of Small-Subunit rRNA Genes in Mixed Microbial Populations via 5′-Nuclease Assays. *Appl. Environ. Microbiol.* **66**, 4605–4614 (2000).
12. U.S. Centers for Disease Control and Prevention. Social Vulnerability Index 2022. https://www.atsdr.cdc.gov/place-health/php/svi/svi-data-documentation-download.html?CDC_AAref_Val=https://www.atsdr.cdc.gov/placeandhealth/svi/data_documentation_download.html.
13. U.S. Census Bureau. 2022 5-Year American Community Survey (ACS). https://www.census.gov/programs-surveys/acs.
14. U.S. Census Bureau. Cartographic Boundary Files. https://www.census.gov/geographies/mapping-files/time-series/geo/cartographic-boundary.html.
15. U.S. Census Bureau. 2020 Census Urban-Rural Classification. https://www.census.gov/programs-surveys/geography/guidance/geo-areas/urban-rural.html (2024).
16. Esri Data and Maps. USA Airports 1,000,000 or more. https://hub.arcgis.com/datasets/esri::usa-airports/about?layer=1.
17. US Department of Homeland Security. Homeland Infrastructure Foundation-Level Data. https://hifld-geoplatform.hub.arcgis.com/ (2024).
18. U.S. Department of Agriculture. 2022 National Agricultural Statistics Service Quick Stats. https://quickstats.nass.usda.gov/.
