# Supplementary material for: Wastewater surveillance reveals patterns of antibiotic resistance across the United States
Source: Nat Commun. 2026 Apr 1;17:4680. doi: 10.1038/s41467-026-71195-4 (PMC13201604; doi:10.1038/s41467-026-71195-4)
Supplement: Supplementary file 3 — Descriptions of Additional Supplementary Files [file 41467_2026_71195_MOESM3_ESM.pdf]

Descriptions of Additional Supplementary Files

Supplementary Data 1

File name: SupplementaryData_Assays

Description: Assays used in this study and expected concentration (high vs low) in wastewater samples from the U.S. based on preliminary testing of seven wastewater samples.

Supplementary Data 2

File name: SupplementaryData_Tables of Locations for AMR Manuscript

Description: Samples provided from each WWTP.
