# Supplementary material for: Wastewater surveillance reveals patterns of antibiotic resistance across the United States
Source: Nat Commun. 2026 Apr 1;17:4680. doi: 10.1038/s41467-026-71195-4 (PMC13201604; doi:10.1038/s41467-026-71195-4)
Supplement: Supplementary file 4 — Supplementary Data 1 [file 41467_2026_71195_MOESM4_ESM.pdf]

nature portfolio

Corresponding author(s): Amy Pickering

Last updated by author(s): 2025-Mar-31

Reporting Summary

Nature Portfolio wishes to improve the reproducibility of the work that we publish. This form provides structure for consistency and transparency in reporting. For further information on Nature Portfolio policies, see our Editorial Policies and the Editorial Policy Checklist.

Statistics

For all statistical analyses, confirm that the following items are present in the figure legend, table legend, main text, or Methods section.

n/a	Confirmed	
☐	☒	The exact sample size (*n*) for each experimental group/condition, given as a discrete number and unit of measurement
☐	☒	A statement on whether measurements were taken from distinct samples or whether the same sample was measured repeatedly
☐	☒	The statistical test(s) used AND whether they are one- or two-sided<br>*Only common tests should be described solely by name; describe more complex techniques in the Methods section.*
☐	☒	A description of all covariates tested
☐	☒	A description of any assumptions or corrections, such as tests of normality and adjustment for multiple comparisons
☐	☒	A full description of the statistical parameters including central tendency (e.g. means) or other basic estimates (e.g. regression coefficient) AND variation (e.g. standard deviation) or associated estimates of uncertainty (e.g. confidence intervals)
☐	☒	For null hypothesis testing, the test statistic (e.g. *F*, *t*, *r*) with confidence intervals, effect sizes, degrees of freedom and *P* value noted<br>*Give P values as exact values whenever suitable.*
☒	☐	For Bayesian analysis, information on the choice of priors and Markov chain Monte Carlo settings
☒	☐	For hierarchical and complex designs, identification of the appropriate level for tests and full reporting of outcomes
☐	☒	Estimates of effect sizes (e.g. Cohen's *d*, Pearson's *r*), indicating how they were calculated

Our web collection on statistics for biologists contains articles on many of the points above.

Software and code

Policy information about availability of computer code

Data collection	QX Manager Software Version 2.2
Data analysis	ArcGIS Pro (version 3.1.1); R (version 4.3.0); caret package (version 7.0-1); dplyr package (version 1.1.2); EnvStats package (version 2.8.1); corrplot package (version 0.92); conover.test package (version 1.1.6); stats package (version 4.3.0); ggplot2 package (version 3.5.1); pheatmap package(1.0.12); https://github.com/sooyeolkim/ww_arg

For manuscripts utilizing custom algorithms or software that are central to the research but not yet described in published literature, software must be made available to editors and reviewers. We strongly encourage code deposition in a community repository (e.g. GitHub). See the Nature Portfolio guidelines for submitting code & software for further information.

Data

Policy information about availability of data

All manuscripts must include a data availability statement. This statement should provide the following information, where applicable:

- Accession codes, unique identifiers, or web links for publicly available datasets
- A description of any restrictions on data availability
- For clinical datasets or third party data, please ensure that the statement adheres to our policy

Data generated in this study on antibiotic resistance gene concentrations in 163 wastewater treatment plants across the United States have been deposited in the Stanford Data Repository under accession code vb318cm9509 (https://purl.stanford.edu/vb318cm9509). The antibiotic prescription data from Epic Cosmos are available under restricted access due to data privacy laws. These data cannot be redistributed or hosted in public repositories to maintain proprietary data

protections. Access can be obtained through affiliation and approval by a Cosmos participating organization or by contacting Epic Cosmos at https://cosmos.epic.com/request-access/. Other secondary data used in this study are publicly available from data sources mentioned in the Methods section

Research involving human participants, their data, or biological material

Policy information about studies with human participants or human data. See also policy information about sex, gender (identity/presentation), and sexual orientation and race, ethnicity and racism.

Reporting on sex and gender	No information regarding sex and gender was used in this study.
Reporting on race, ethnicity, or other socially relevant groupings	Census data for race and ethnicity was used to assess correlation with antibiotic resistance gene prevalence. Proportion of people who speak limited English (defined as Proportion of persons age 5+ years who speak English "less than well") was used as an indication of recent immigration and travel. Proportion of certain race were defined as: proportion of the population that is Black/African American, not Hispanic or Latino; proportion of the population this is Hispanic or Latino; and proportion of the population that is Asian, not Hispanic or Latino.
Population characteristics	Census data for various socioeconomic variables and age (under 18 or over 65) was considered.
Recruitment	No recruitment was done as this study involved only wastewater monitoring of targets and use of data from the census and Epic Cosmos.
Ethics oversight	Ethics oversight was not needed as this study only included environmental samples.

Note that full information on the approval of the study protocol must also be provided in the manuscript.

Field-specific reporting

Please select the one below that is the best fit for your research. If you are not sure, read the appropriate sections before making your selection.

☐ Life sciences ☐ Behavioural & social sciences ☒ Ecological, evolutionary & environmental sciences

For a reference copy of the document with all sections, see nature.com/documents/nr-reporting-summary-flat.pdf

Ecological, evolutionary & environmental sciences study design

All studies must disclose on these points even when the disclosure is negative.

Study description	In this study, we use dPCR to quantitatively measure the abundance of 11 clinically relevant ARGs that confer resistance to beta-lactams (CMY, CTX-M, KPC, NDM, mecA, OXA, TEM, VIM), colistin (mcr-1), tetracycline (tetW), and vancomycin (vanA) from 163 wastewater sites to generate a cross-sectional data set of the U.S.
Research sample	The research sample is wastewater solids from wastewater treatment plants. We chose to sample solids since previous research has shown that pathogens are effectively detected from wastewater solids. In addition, we were leveraging an on-going national surveillance effort that sampled wastewater solids.
Sampling strategy	Wastewater treatment plant staff provided either “grab” samples from the primary clarifier or 24-hour composite samples from the headworks. The “grab” samples in this case are solids collected in the primary clarifier over 1-8 hours, representing composite community wastewater solids. No sample calculation was conducted since this study leverages current on-going national surveillance efforts for viruses.
Data collection	The ARG data was collected by Dorothea Duong using QX600 Droplet Digital PCR system. The 16S rRNA data was collected by Sooyeol Kim and Alessandro Zulli using the QX200 Droplet Digital PCR system.
Timing and spatial scale	Samples were collected between May 7, 2024 and May 23, 2024 from 163 wastewater treatment plants in the U.S. Most of the wastewater treatment plants were sampled over a week and 1-3 samples were collected. The samples were treated as a replicate and the concentrations obtained over the collection period was averaged to capture variation in concentration over a short period of time.
Data exclusions	Data points that were considered as significant outliers by the Rosner's test for 16S rRNA or 3 standard deviations away in distribution of the ARG were excluded when using the data with secondary data sets to limit the effect of these outliers in analysis.
Reproducibility	Our choice to treat samples collected over a week was our effort to increase reproducibility of the study.
Randomization	No randomization was conducted and all samples were processed.
Blinding	Samples were assigned IDs before being processed and then rematched with their location information after.

Did the study involve field work? ☐ Yes ☒ No

Reporting for specific materials, systems and methods

We require information from authors about some types of materials, experimental systems and methods used in many studies. Here, indicate whether each material, system or method listed is relevant to your study. If you are not sure if a list item applies to your research, read the appropriate section before selecting a response.

Materials & experimental systems

n/a	Involved in the study
☒	☐ Antibodies
☒	☐ Eukaryotic cell lines
☒	☐ Palaeontology and archaeology
☒	☐ Animals and other organisms
☒	☐ Clinical data
☒	☐ Dual use research of concern
☒	☐ Plants

Methods

n/a	Involved in the study
☒	☐ ChIP-seq
☒	☐ Flow cytometry
☒	☐ MRI-based neuroimaging

Plants

Seed stocks	*Report on the source of all seed stocks or other plant material used. If applicable, state the seed stock centre and catalogue number. If plant specimens were collected from the field, describe the collection location, date and sampling procedures.*
Novel plant genotypes	*Describe the methods by which all novel plant genotypes were produced. This includes those generated by transgenic approaches, gene editing, chemical/radiation-based mutagenesis and hybridization. For transgenic lines, describe the transformation method, the number of independent lines analyzed and the generation upon which experiments were performed. For gene-edited lines, describe the editor used, the endogenous sequence targeted for editing, the targeting guide RNA sequence (if applicable) and how the editor was applied.*
Authentication	*Describe any authentication procedures for each seed stock used or novel genotype generated. Describe any experiments used to assess the effect of a mutation and, where applicable, how potential secondary effects (e.g. second site T-DNA insertions, mosiacism, off-target gene editing) were examined.*
